# Effectiveness of a brief group behavioral intervention for common mental disorders in Syrian refugees in Jordan: A randomized controlled trial

**DOI:** 10.1371/journal.pmed.1003949

**Published:** 2022-03-17

**Authors:** Richard A. Bryant, Ahmad Bawaneh, Manar Awwad, Hadeel Al-Hayek, Luana Giardinelli, Claire Whitney, Mark J. D. Jordans, Pim Cuijpers, Marit Sijbrandij, Peter Ventevogel, Katie Dawson, Aemal Akhtar, on behalf of the STRENGTHS Consortium

**Affiliations:** 1 University of New South Wales, Sydney, Australia; 2 Westmead Institute of Medical Research, Sydney, Australia; 3 Jordan Country Office, International Medical Corps, Amman, Jordan; 4 International Medical Corps, Washington DC, United States of America; 5 War Child, Amsterdam, the Netherlands; 6 University of Amsterdam, Amsterdam, the Netherlands; 7 Vrije Universiteit, Amsterdam, the Netherlands; 8 United Nations High Commissioner for Refugees, Geneva, Switzerland; Johns Hopkins Bloomberg School of Public Health, UNITED STATES

## Abstract

**Background:**

Common mental disorders are frequently experienced by refugees. This study evaluates the impact of a brief, lay provider delivered group-based psychological intervention [Group Problem Management Plus (gPM+)] on the mental health of refugees in a camp, as well as on parenting behavior and children’s mental health.

**Methods and findings:**

In this single-blind, parallel, randomized controlled trial, 410 adult Syrian refugees (300 females, 110 males) in Azraq Refugee Camp (Jordan) were identified through screening of psychological distress (≥16 on the Kessler Psychological Distress Scale) and impaired functioning (≥17 on the WHO Disability Assessment Schedule). Participants were randomly allocated to gPM+ or enhanced usual care (EUC) involving referral information for psychosocial services on a 1:1 ratio. Participants were aware of treatment allocation, but assessors were blinded to treatment condition. Primary outcomes were scores on the Hopkins Symptom Checklist-25 (HSCL; depression and anxiety scales) assessed at baseline, 6 weeks, and 3 months follow-up as the primary outcome time point. It was hypothesized that gPM+ would result in greater reductions of scores on the HSCL than EUC. Secondary outcomes were disability, posttraumatic stress, personally identified problems, prolonged grief, prodromal psychotic symptoms, parenting behavior, and children’s mental health. Between October 15, 2019 and March 2, 2020, 624 refugees were screened for eligibility, 462 (74.0%) screened positive, of whom 204 were assigned to gPM+ and 206 to EUC. There were 168 (82.4%) participants in gPM+ and 189 (91.7%) in EUC assessed at follow-up. Intent-to-treat analyses indicated that at follow-up, participants in gPM+ showed greater reduction on HSCL depression scale than those receiving EUC (mean difference, 3.69 [95% CI 1.90 to 5.48], *p* = .001; effect size, 0.40). There was no difference between conditions in anxiety (mean difference −0.56, 95% CI −2.09 to 0.96; *p* = .47; effect size, −0.03). Relative to EUC, participants in gPM+ had greater reductions in severity of personally identified problems (mean difference 0.88, 95% CI 0.07 to 1.69; *p* = .03), and inconsistent disciplinary parenting (mean difference 1.54, 95% CI 1.03 to 2.05; *p* < .001). There were no significant differences between conditions for changes in PTSD, disability, grief, prodromal symptoms, or childhood mental health outcomes. Mediation analysis indicated the change in inconsistent disciplinary parenting was associated with reduced attentional (β = 0.11, SE .07; 95% CI .003 to .274) and internalizing (β = 0.08, SE .05; 95% CI .003 to 0.19) problems in children. No adverse events were attributable to the interventions or the trial. Major limitations included only one-quarter of participants being male, and measures of personally identified problems, grief, prodromal psychotic symptoms, inconsistent parenting behavior, and children’s mental health have not been validated with Syrians.

**Conclusions:**

In camp-based Syrian refugees, a brief group behavioral intervention led to reduced depressive symptoms, personally identified problems, and disciplinary parenting compared to usual care, and this may have indirect benefits for refugees’ children. The limited capacity of the intervention to reduce PTSD, disability, or children’s psychological problems points to the need for development of more effective treatments for refugees in camp settings.

**Trial registration:**

Prospectively registered at Australian and New Zealand Clinical Trials Registry: ACTRN12619001386123.

## Abstract 2

Richard A. Bryant and colleagues evaluate effects of a lay provider-delivered intervention on adult Syrian refugees’ mental health, parenting behavior, and their children’s mental health.

## Author summary

**Why was this study done?:**

- There are approximately 80 million refugees and forcibly displaced people today, and they are at greater risk for common mental disorders.
- Most countries hosting refugees lack the health resources to address these mental health problems.
- **In response to this situation, the World Health Organization has developed a brief psychological intervention that can be delivered by trained lay workers that aims to reduce psychological distress following adversity.**
- **To our knowledge, this study was the first to test the effectiveness of the Group Problem Management Plus (gPM+) program in distressed refugees in a refugee camp.**

**What did the researchers do and find?:**

- This study screened adult Syrian refugees in a camp in in Jordan, and subsequently randomized 410 adult refugees who displayed distress and impaired functioning to either 5 individual sessions of gPM+ or enhanced usual care (EUC).
- **Independent assessments indicated that 3 months following treatment, refugees who received gPM+ reported significantly less depression but not anxiety, with a moderate effect relative to EUC.**
- **gPM+ also led to greater reductions inconsistent disciplinary parenting, and this was associated with greater reductions in attentional and internalizing problems in refugees’ children.**

**What do these findings mean?:**

- This study suggests that mental health nonspecialists can be briefly trained in gPM+, and they can deliver this intervention in a way that it can improve mental health of refugees.
- This brief intervention has the potential to scale up mental health services for refugees in settings that lack adequate health resources, and this may have downstream benefits for refugees’ children.

## Introduction

Worldwide, nearly 80 million people are forcibly displaced by war and conflict, with over 26 million formally registered refugees and many more who are not registered [1]. People exposed to this form of adversity are at greater risk for common mental disorders, including anxiety, depression, and posttraumatic stress disorder (PTSD) [2]. This is not surprising considering the exposure to violence, traumatic death, forced separation from family and social networks, poverty, and hardship that people in these situations endure. Refugees displaced from war-affected settings are often hosted in directly adjacent low- and middle-income countries (LMICs) that lack sufficient resources to provide adequate mental health services. The recent Lancet Commission on global mental health highlighted the marked gap between mental health need and provision of services in LMICs [3]. The major barriers to providing evidence-based mental health programs in LMICs include the lack of mental health specialists, most treatment programs being disorder-specific, which results in health providers needing to master multiple programs, and lengthy programs that are costly for health services and demanding on recipients [4].

In response to these challenges, a range of task-shifting approaches has been developed in which nonspecialists are trained and supervised to deliver mental health programs. Meta-analyses indicate that this approach can be effective [5–7]. The World Health Organization has developed one such program, Problem Management Plus (PM+) [8] that is a 5-session program that adopts a transdiagnostic approach to reduce common mental disorders by teaching skills in arousal reduction, problem-solving, behavioral activation, and accessing social support [9]. This program has been shown to be effective in large trials in both individual (PM+) [10,11] and group (gPM+) [12] formats in communities affected by adversity. One of the major challenges for advancing the generalizability of gPM+ to populations most affected by prior trauma and ongoing adversity is to determine its effectiveness in refugee camp settings in which people have significant restrictions on their movements, choices, and capacity to exert control over their futures.

Central to the issue of global mental health is the psychological well-being of children. Although the efficacy of gPM+ and other task-shifting programs have been shown, there is a scarcity of effective programs that address the needs of children [13]. This is a particularly an urgent issue for refugee children and youth, who represent more than half of refugees in the world today [1]. Many reports indicate that refugee youth experience high rates of psychological distress [14–16]. One possible means to improve the psychological well-being of refugee youth is by improving the psychological health of their parents or caregivers [17,18]. There is evidence that refugees’ mental health is associated with the psychological status of their children, and it appears this association is partly attributed to their parenting behavior, which, in turn, affects the psychological well-being of their children [19–22]. For example, one population-based study found that refugees’ PTSD severity was associated with harsh parenting, which was, in turn, associated with worse psychological problems in their children [19]. This convergent evidence raises the possibility that reducing common mental disorders in refugees may improve their parenting behavior, which may have a downstream beneficial effect on the psychological health of their children; this possibility is supported by meta-analysis indicating that psychotherapy for depressed mothers has beneficial effects of their children’s mental health [23].

To address the significant mental health needs of refugees, and particularly those in refugee camp settings, we conducted a randomized controlled trial (RCT) of gPM+ to adult Syrian refugees in a camp in Jordan and compared this program to enhanced usual care (EUC). This trial focused on refugees in a camp because of the limited evidence regarding scalable psychological programs for refugees in camps. Worldwide, approximately one-quarter of refugees reside in camps [24]. It is important to test the capacity of gPM+ in refugees who are in camp settings because there can be specific stressors in refugee camps, as well as some limitations that may limit the extent to which refugees can use the skills taught in gPM+. For example, refugees in camps may have restricted movement, limited employment opportunities, and separation from family that can compound psychological difficulties and hamper the extent to which problem management strategies may be employed [25–27]. This trial also investigated the potential impact of gPM+ on refugees’ parenting behavior, and also how this may benefit their children’s mental health. It was hypothesized that gPM+ would reduce anxiety and depression, disability, posttraumatic stress, personally identified problems, prolonged grief, prodromal psychotic symptoms, as well as improving parenting behaviors and children’s mental health, relative to EUC at the follow-up assessment.

## Method

### Study design

This two-arm, single-blind RCT was conducted in Azraq Refugee Camp in Jordan. There are over 650,000 Syrian refugees registered with the UNHCR in Jordan; however, the government estimates there are more than 1.4 million Syrians currently residing in Jordan [28]. Azraq Refugee Camp hosts approximately 36,000 Syrian refugees, of which 60% are youth. The study was conducted in collaboration with International Medical Corps (IMC) Jordan, who was the local implementer in Jordan. The project was prospectively registered (Australian and New Zealand Clinical Trials Registry, no. 12619001386123) and ethically approved by the Institutional Review Board at the King Hussein Cancer Centre in Jordan and the University of New South Wales Human Research Ethics Committee. The trial protocol is available in S1 Text and online [29]. This study is reported as per the Consolidated Standards of Reporting Trials (CONSORT) guideline (S1 Checklist).

### Participants

Participants comprised refugees in Azraq Refugee Camp and inclusion criteria were as follows: (a) aged ≥18 years; (b) scores ≥16 on the Kessler Psychological Distress Scale (K10 [30]); (c) Arabic-speaking; (d) scores ≥17 on the WHO Disability Assessment Schedule 2.0 (WHODAS [31]); and (e) had a child or dependent living in the household aged 10 to 16 years. The K10 is a 10-item questionnaire that assesses psychological distress, with a range of 10 to 50; a cutoff of 16 has been shown to indicate psychological distress [32]. The K-10 has been validated in Syrian populations [26], and the recommended cutoff of 16 has been successfully used in refugee populations [33]. This measure was employed as a brief screener to permit rapid screening of refugees with psychological distress. The WHODAS is a 12-item questionnaire that assesses general functioning, with a possible total score of 48; following prior trials of gPM+ [10,11], a cutoff of 17 was used to identify functional impairment. Exclusion criteria were as follows: (a) significant cognitive or neurological impairment; (b) acute medical conditions; (c) severe mental disorders (e.g., psychotic or substance abuse disorders); and (d) acute risk of suicide. Exclusion criteria were assessed by structured questions pertaining to each criterion described in the WHO PM+ manual [8]. Refugees meeting exclusion criteria were referred to specialized services in the camp. Recruitment was conducted by Arabic-speaking assessors through door-to-door screening of consecutive caravans; to reduce contamination of the interventions, only 1 adult per caravan was invited to participate in the study. It was considered that there would likely not be significant contamination between neighboring caravans because socialization did not adhere to caravan proximity. The invitation was initially extended to the person who answered the door, and if they declined, the offer was made to another adult living in the caravan. Informed consent involved 2 steps: (1) consent to participate in the screening; and (2) participants who screened positive were invited to provide their consent to participate in the trial. Participants completed a written consent form, and those who were illiterate provided witnessed oral consent, in line with recommendations from WHO [34]. Additionally, caregivers were asked to provide written consent for participation for one of their children whom the caregiver nominated between the ages of 10 and 16 years to be assessed; additionally, verbal assent was obtained from the child. Children were then approached to obtain assent to complete the Pediatric Symptoms Checklist (PSC-35) [35] during the pre- and post-assessments; children’s assent was not a requirement for participation of their caregiver.

### Randomization and masking

Participants were randomly allocated (on a 1:1 ratio) to either a 5-week gPM+ intervention or EUC. Randomization was conducted at the UNSW Australia by staff who were independent of the trial using computerized software that generated random number sequences. Allocation concealment was ensured by keeping the treatment assignments in sequentially numbered, opaque, sealed envelopes that informed the trial coordinator in Jordan on assignment to gPM+ or EUC. Masking participants and facilitators was not achieved because allocation to the respective condition was transparent. Assessors who identified participants, enrolled participants into the trial, and conducted all assessments were masked to treatment condition allocation. Assessors were located separately from the refugee camp, and at no point did they interact with group facilitators. At the commencement of each assessment assessors instructed participants to not inform them about their allocated condition.

### Procedures

Prior to the trial, the translation and cultural adaptation of gPM+ was reviewed in adaptation workshops with Syrian representatives and local healthcare providers, and a pilot trial to determine logistic issues that needed to be addressed in the camp prior to the current trial [36]. The measures and intervention were adapted for cultural appropriateness in terms of language, metaphors, and context. The adaptation process was conducted via free list interviews, key informant interviews, focus groups, and adaptation workshops; the full details of the adaptation procedures are described elsewhere [37].

The gPM+ program followed the WHO group program [38]. The gPM+ intervention comprised 5 weekly 2-hour group sessions (8 to 10 people per group that were gender specific). Session 1 established group rules, psychoeducation, and a stress management strategy. Session 2 focused on problem-solving strategies. Session 3 provided instructions in behavioral activation. Session 4 focused on strategies in accessing social supports. Finally, Session 5 reviewed all strategies and addressed relapse prevention options. In addition, Sessions 2 to 4 reviewed progress on previously taught strategies. The focus in the group sessions were to teach the participants skills in managing the stressors faced in the camp, and group discussion was facilitated to encourage group members to provide input on problem-solving the common challenges that participants experienced on a daily basis.

The groups were conducted by 2 facilitators. The facilitators held a bachelor’s degree in social sciences or a related health discipline, were proficient in Arabic, but had no prior experience in delivering psychosocial programs. The facilitators received 8 days of training in the delivery of gPM+, as well as basic counseling and group facilitation skills. Following training, the gPM+ providers were required to complete 2 practice groups, as a lead facilitator and as a cofacilitator, under close supervision. In addition, a local supervisor (HAH) who worked within the camp provided weekly supervision throughout the trial. The local supervisor also received fortnightly supervision by a primary trainer of gPM+ in Sydney, Australia (KD) via Skype calls. To evaluate treatment fidelity, 20% of gPM+ sessions were attended by the supervisor, who used a checklist to ensure that all gPM+ components were delivered. Each strategy was checked by the supervisor as present/absent, and whether it was delivered satisfactorily or not. Fidelity checks via recording of sessions were not possible because of concerns from refugees that recorded information disclosed during sessions may be accessed by government or Syrian authorities and used against them or their families.

Participants randomized to EUC received a visit to their caravan from IMC staff and given specific information about their services in the camp that could assist with the problems identified in the assessment. This information included organizations providing services for mental health problems, as well as health, parenting, and vocational training. Sessions were approximately 15 minutes in duration. If participants in EUC displayed severe psychiatric problems (e.g., psychosis or suicidality) during the feedback sessions that were not reported in the initial assessment that required immediate attention, they were referred to IMC mental health clinics in the camp for further intervention. If risk of harm was determined, participants were referred to the National Centre for Mental Health in Amman.

### Outcomes

The primary outcome was the total score for anxiety and depression, respectively, measured with the Hopkins Symptom Checklist-25 (HSCL-25) [39]. The HSCL consists of 25 questions that are rated on a 4-point scale (1 = not at all, 4 = extremely), with higher total scores reflecting more severe anxiety and depression. The HSCL has been validated across many cultures, including in Arabic contexts [40]; recommended cutoffs for probable caseness of anxiety and depression on the Arabic version of the HSCL relative to structured clinical interview is 2.0 and 2.1, respectively [40]. The internal consistency of the HSCL in the current sample was robust for the anxiety (0.79) and depression (0.84) scales, respectively.

Secondary outcomes were functional impairment, PTSD symptoms, personally identified problems, prolonged grief symptoms, prodromal psychotic symptoms, parenting behavior, and children’s self-reported mental health. Functional impairment was assessed using the 12-item WHODAS 2.0, with each item scored of 4-point scale and higher scores indicating more severe disability. The WHODAS has good psychometric properties across many countries [31], and the internal consistency in the current study was 0.82. PTSD symptoms were measured using the PCL-5 [41], which is a 20-item checklist corresponding with the 20 DSM-5 PTSD symptoms. Items are rated on a 5-point scale, with higher scores indicating more severe PTSD. A validity study of Syrian and Iraqi refugees observed that a score of 23 is the optimal cutoff to indicate probable PTSD diagnosis [42]. Personally identified problems were assessed with the Psychological Outcome Profiles (PSYCHLOPS) [43], which address 3 domains (main problems experienced, 2 questions), functioning (1 question), and well-being (1 question). Each response is scored on a 6-point scale, with a possible total score of 20 on which higher scores indicate greater problem severity. The PSYCHLOPS has been validated repeatedly in global mental health [44,45] and has been shown to be sensitive to change in prior PM+ and gPM+ trials [10–12]. Prolonged grief symptoms were assessed using the PG-13 [46], which is a 13-item self-report measure that indexes the core symptoms of prolonged grief disorder (PGD); in current study, the internal consistency was 0.86. Eleven items are rated on a 5-point scale and 2 items on a 2-item scale, providing a possible total score of 57 with higher score reflecting more severe grief. Prodromal psychotic symptoms were assessed using the Prodromal Questionnaire-16 (PQ-B [47]). The PQ-B comprises 16 true or false items about early signs of psychosis and asks about levels of distress experienced for the endorsed items on a 4-point scale; higher scores indicate worse prodromal symptoms. Respondents who endorse ≥6 items are considered to be at risk for developing psychosis [47]; the internal consistency was 0.82. Parenting behavior was assessed using the Alabama Parenting Questionnaire-42 (APQ) [48]. The APQ measures 5 major parenting constructs: (i) involvement (10 items); (ii) poor supervision and monitoring (10 items); (iii) positive parenting (6 items); (iv) inconsistent discipline (6 items); and (v) corporal punishment (3 items). Each item is scored on a 5-point scale, with higher scores indicating greater strength of the relevant subscale. Psychological distress in children of participants was assessed using the youth-reported version of the Pediatric Symptoms Checklist (PSC), which has been validated in 6- to 16-year-olds [35]. The PSC comprises 35 items rated on a 3-point scale and yields a total score, as well as 3 subscale scores of attentional (5 items), internalizing (5 items), and externalizing (7 items) problems. Higher scores indicate more severe difficulties in the respective domain. Additionally, at baseline, prior exposure to traumatic events was assessed using an adapted form of published Traumatic Events Checklists [49,50]; this 27-item dichotomously scored checklist provided a potential score of 27, with higher scores indicating greater exposure to traumatic events. Ongoing stressors were assessed at baseline using the 17-item Post-Migration Living Difficulties checklist [51], which assesses the extent to which post-migration challenges concern the respondent over the prior 12 months; items were rated on a 5-point scale, with items scores ≥2 (a moderately serious problem) were defined as the stressor being present, with higher scores indicating more ongoing stressors. This scale has previously been used in Arabic-speaking refugees [52].

All assessments were conducted by Arabic-speaking Jordanians, who received 4 days of training in research ethics, the assessment battery, and general interviewing techniques, and psychological first aid in order to allow them to manage any distressed participants during an assessment. Assessments were conducted on portable tablets to ensure that data could be reliably collected and uploaded. On the basis that a proportion of participants were inadequately literate in Arabic, assessors verbally administered the questions and assessors entered participants’ responses on the tablets.

### Statistical analyses

We based our power analysis on prior trials of gPM+ [10] and aimed for a medium Cohen’s *d* effect size of 0.4 in the gPM+ group at 3-month follow-up (the primary outcome time point). Power calculations suggest a minimum sample size of 133 participants per group (power = 0.90, *a* = 0.05, two-sided). Taking into account an expected 35% attrition at 3-month follow-up, we aim to include a total number of 410 participants (205 in gPM+ and 205 in EUC).

Analyses focused primarily on intent-to-treat analysis. Linear mixed models were used to study differential effects of each treatment condition because this method allows the number of observations to vary between participants. Further, this statistical approach handles missing data by including all available data and using maximum likelihood estimation methods, which makes valid inferences under the assumption of data missing at random [53]. Fixed (intervention, time of assessment) effects and their interactions were entered in the unstructured models, which provided an index of the relative effects of the treatments; time of assessment included baseline, posttreatment, and 3-month follow-up. Fixed effects parameters were tested with the Wald test (*t* test, *p* < .05, two-sided) and 95% confidence intervals (no Bonferroni adjustment was made). Analyses focus on the primary (HSCL) and secondary (WHODAS, PCL, PSYCHLOPS, PG-13, PQB, APQ, and PSC scores) outcomes, with the main outcome point being the 3-month follow-up. Missing data were assumed to be random on the basis that participants completing the 3-month assessment and those who were missing did not differ in terms of age, education level, trauma exposure, or primary outcome measures at baseline (S1 Table). Further, there were only 43 (10.5%) of cases in which there was no follow-up data on the primary outcome measures. To assess the robustness of this statistical approach, we conducted subsequent analyses including only participants who completed the 3-month follow-up. Further, in recognition of the variable impacts of prior traumatic experiences and ongoing stressors experienced by participants, analyses were repeated adjusting the models using baseline scores of the Traumatic Events Checklist and the Post-Migration Living Difficulties as covariates. To determine the effectiveness of the intervention on refugees with probable clinically significant problems, we also conducted sensitivity analyses focusing on refugees who presented with probable anxiety or depression on the HSCL (defined as mean item score ≥2 on anxiety or ≥2.1 on depression subscales). We also conducted nonplanned analyses on the minimally important difference for outcomes by comparing the proportions of participants in each treatment arm showing changes of more than 0.5 SDs [54].

To explore the potential role of gPM+ on parenting and children’s mental health, an exploratory secondary mediation analysis was conducted by assessing the direct and indirect effects of intervention arm on change in APQ subscale scores from baseline to 3-month follow-up, and change in PSC subscale scores. The proposed mediation model was examined using the PROCESS macro (Version 3.15; [55]) for SPSS (Version 27), PROCESS Model 4, with 5,000 bootstrapped samples. This model examined the relationship between intervention arm on changes in child mental health (PSC subscale scores), with changes in parenting behaviors (APQ subscale scores) as the mediators. All analyses were overseen by an independent statistician who was blind to treatment condition.

## Results

Participants were enrolled between October 14, 2019 and March 2, 2020, and the final follow-up assessments were conducted on July 6, 2020. There were 1,377 caravans approached, and this resulted in 624 refugees agreeing to be screened. There were 462 refugees meeting entry criteria, of whom 410 proceeded to randomization; 204 refugees were randomized to gPM+ and 206 to EUC. The primary outcome assessment at 3 months was conducted for 168 (82.4%) participants in gPM+ and 189 (91.7%) in EUC. This level of attrition was within the projected 35% margin on which the power analysis was calculated. The attrition was much lower than we expected, possibly because of unfolding events in Syria that may have caused many refugees to be reluctant to return home. Interestingly, there was 10% greater attrition in the gPM+ arm than EUC at the 3-month follow-up, possibly reflecting fatigue in participating in the trial. No adverse events were attributable to the interventions or the trial.

**Fig 1:**
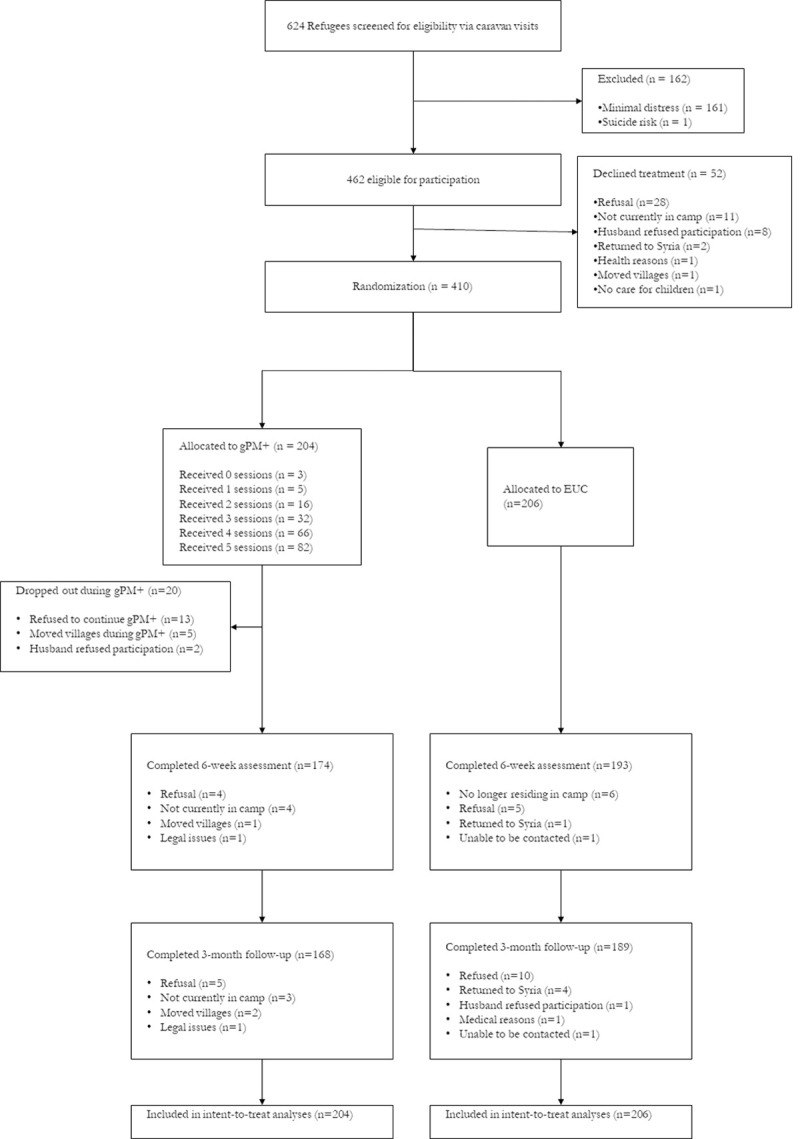
Flow diagram of progress through phases of a randomized trial comparing the group problem management plus intervention vs EUC in Syrian refugees in Azraq Refugee Camp, Jordan. Five participants completed the 3-month follow-up who did not complete the posttreatment assessment. EUC, enhanced usual care; gPM+, Group Problem Management Plus.

Participants who were lost at follow-up did not differ from those who were retained in terms of age, education level, trauma exposure, or baseline scores on any outcome measures with the exception of those who were lost to follow-up having had higher positive parenting and parental involvement scores (S2 Table). The flowchart of participant recruitment and retention is reported in Fig 1. Sample characteristics are presented in Table 1. The mean age of participants was 40.03 years (SD 6.95), most were female (300 [70.2%] females, 110 [29.8%] males), and the mean time since leaving their home in Syria was 5.89 years (SD 1.67; range: 1 to 9 years). The demographic characteristics were not significantly different between the 2 treatment arms. Notably, the participants reported common exposure to potentially traumatic events, including danger during fleeing from Syria (84.6%), direct exposure to war (69.3%), accidents (66.3%), disasters (31.7%), serious injury (23.9%), forced separation from family (21.5%), and murder of someone close to them (11.7%) (see Table 2). In terms of current stressors, many refugees reported poverty (90.2%), poor work conditions (83.4%), loneliness (70.2%), worry for family in Syria (77.3%), and poor healthcare (67.1%). The sample included 267 (65.1%) with probable depression, 321 (78.3%) with probable anxiety disorder, and 138 refugees (61.5%) who reported probable PTSD.

**Table 1:** Participant characteristics.

	Total (*n =* 410)	gPM+ (*n* = 204)	EUC (*n* = 206)
Female, n (%)	300 (73.2)	145 (71.1)	155 (75.2)
Age, years (SD)	40.03 (6.95)	39.38 (6.71)	40.68 (7.13)
Married, n (%)			
Single	0 (0)	0 (0)	0 (0)
Married	376 (91.7)	188 (92.2)	35 (91.3)
Separated	16 (3.9)	8 (3.9)	8 (3.9)
Divorced	5 (1.2)	3 (1.5)	2 (1.0)
Widowed	13 (3.2)	5 (2.5)	8 (3.9)
Education, n (%)			45 (21.8)
None	102 (24.9)	56 (27.5)	46 (22.3)
Basic certificate (10 years of eduction)	233 (56.8)	113 (55.4)	120 (58.3)
Technical trade certificate	32 (7.8)	17 (8.3)	15 (7.3)
Secondary education (12 years of education)	34 (8.3)	14 (6.8)	20 (9.7)
University degree	9 (2.2)	4 (2.0)	5 (2.4)
Time since leaving Syria			
Less than 4 years	95 (23.2)	45 (22.1)	50 (24.3)
5–6 years	162 (39.5)	80 (39.2)	82 (39.8)
7–9 years	153 (37.3)	79 (38.7)	74 (35.9)
Probable Depression	267 (65.1)	140 (68.6)	127 (61.7)
Probable Anxiety	321 (78.3)	160 (78.4)	161 (78.2)
Probable PTSD	252 (61.5)	120 (58.8)	132 (64.1)

EUC, enhanced usual care; gPM+, Group Problem Management Plus; PTSD, posttraumatic stress disorder.

Probable Depression ≥2.1 on the Hospital Anxiety Depression Scale: Depression Scale. Probable Anxiety ≥2.0 on the Hospital Anxiety Depression Scale: Anxiety Scale. Probable PTSD ≥23 on the Postraumatic Stress Disorder Checklist 5.

**Table 2:** Frequencies and percentages of rates of potentially traumatic exposures and current stressors.

Potentially traumatic exposure N (%)	Total (*n =* 410)	gPM+ (n = 204)	EUC (*n* = 206)	Current stressors N (%)	Total (*n* = 410)	gPM+ (*n* = 204)	EUC (*n* = 206)
Disaster	126 (30.7)	65 (31.9)	61 (29.6)	Communication difficulties	99 (24.1)	49 (24.0)	50 (24.3)
Serious injury	98 (23.9)	53 (26.0)	45 (21.8)	Discrimination	174 (42.4)	97 (47.5)	77 (37.4)
Serious accident	272 (66.3)	137 (67.2)	135 (65.5)	Ethnic conflict	40 (9.8)	25 (12.5)	15 (7.3)
Serious illness	139 (33.9)	72 (35.3)	67 (32.5)	Family separation	261 (63.7)	130 (63.7)	131 (63.6)
Danger in flight	347 (84.6)	179 (87.7)	168 (81.6)	Worry for family	317 (77.3)	154 (74.8)	163 (79.9)
Physical assault	43 (10.5)	24 (11.8)	19 (9.3)	Cannot return to Syria in emergency	241 (58.8)	120 (58.3)	121 (59.3)
Imprisoned	61 (14.9)	35 (17.2)	26 (12.6)	Poor work conditions	342 (83.4)	168 (81.6)	174 (85.3)
Forced separation from family	347 (84.6)	44 (21.6)	44 (21.4)	Migration problem	62 (15.1)	31 (15.0)	31 (15.2)
War exposure	284 (69.3)	144 (70.6)	140 (68.0)	Lack of healthcare	275 (67.1)	137 (66.5)	138 (67.6)
Lack of food/water	293 (71.5)	149 (73.0)	144 (69.9)	Poverty	370 (90.2)	187 (90.8)	183 (89.7)
Unnatural death of family/friend	83 (20.2)	43 (21.1)	40 (19.4)	Loneliness	288 (70.2)	154 (75.5)	134 (65.0)
Murder of friend/family	48 (11.7)	24 (11.8)	24 (11.7)	Poor accommondation	55 (13.4)	32 (15.7)	23 (11.2)
Disappearance of family/friend	71 (17.3)	38 (18.6)	33 (16.0)	Illness with no healthcare	245 (59.8)	124 (60.8)	121 (58.7)
Torture	36 (8.8)	19 (9.2)	17 (8.3)	No financial assistance	300 (73.2)	147 (72.1)	153 (74.3)

EUC, enhanced usual care; gPM+, Group Problem Management Plus.

The mean number of gPM+ sessions attended was 3.96 (SD 1.14), with 180 (88%) participants attending at least 3 sessions. Twenty-three gPM+ sessions were observed throughout the study to evaluate the intervention fidelity, 22 (95.7%) of which were assessed to be satisfactory. Two-thirds (126; 66.7%) of refugees in EUC reported consulting a health professional in the camp during the period of the trial; more than half of these consultations were to the camp medical officers (58.2%). Two participants in gPM+ disclosed their allocation during the posttreatment assessment, and one participant in gPM+ disclosed their allocation at the follow-up assessment.

The primary and secondary outcomes at each time point are presented in Table 3. In terms of primary outcomes, at the 3-month follow-up assessment, the gPM+ had a greater reduction in HSCL-Depression scores than those in the EUC group (adjusted mean difference 3.69, 95% CI 1.90 to 5.48; *p* < .001; effect size, 0.40). There were more participants in the gPM+ arm (102; 50.0%) relative to EUC (89; 43.2%) achieving a minimally important difference in depression between baseline and follow-up (χ^2^ = 6.67, *p* = .03) (S2 Table). There was no difference between treatment arms in change in anxiety levels (adjusted mean difference −0.56, 95% CI −2.09 to 0.96; *p* = .47; effect size, −0.03) at the 3-month follow-up.

**Table 3:** Summary statistics and results from mixed model analysis of primary and secondary outcomes.

		Descriptive statistics		Mixed model analysis		
Primary and secondary outcomes	Visit	Intervention (*n =* 204)	EUC (*n* = 206)	Difference in LS mean (95% CI)	*p*-value	Effect size^a^
		Estimated mean (SE)	Estimated mean (SE)			
HSCL-25 Depression	Baseline (*n* = 410)	36.57 (.63)	35.15(.63)			
	6-week (*n =* 367)	29.98 (.73)	32.44 (.70)	3.89 (1.98, 5.80)	.001	0.43
	3 months (*n* = 357)	29.04 (.72)	31.30 (.72)	3.69 (1.90, 5.48)	.001	0.40
HSCL-25 Anxiety	Baseline (*n* = 410)	24.81 (.43)	25.00 (.43)			
	6-week (*n* = 367)	20.39 (.50)	21.93 (.48)	−1.34 (−0.08, 2.77)	.06	−0.09
	3 months (*n* = 357)	20.03 (.50)	19.65 (.48)	−.56 (−2.09, 0.96)	.47	−.03
WHODAS	Baseline (*n* = 410)	23.70 (.36)	23.76 (.35)			
	6-week (*n* = 366)	13.99 (.60)	16.20 (.57)	0.48 (−1.18, 2.14)	.57	0.19
	3 months (*n* = 357)	14.97 (.56)	16.81 (.53)	−1.21 (−2.80, 0.37)	.13	0.48
PCL-5	Baseline (*n* = 410)	25.98 (1.02)	26.72 (1.02)			
	6-week (*n* = 366)	16.12 (1.05)	17.58 (1.01)	0.72 (−2.60, 4.03)	0.67	0.10
	3 months (*n* = 357)	10.31 (1.02)	10.37 (.97)	−0.68 (−4.01, 2.66)	0.69	−0.09
PSYCHLOPS	Baseline (*n* = 410)	16.48 (.27)	15.72 (.27)			
	6-week (*n* = 365)	13.34 (.36)	13.67 (.35)	1.09 (0.19, 1.98)	0.02	0.57
	3 months (*n =* 357)	13.46 (.34)	13.59 (.33)	0.88 (0.07, 1.69)	.03	0.46
PG-13	Baseline (*n* = 234)	27.97 (.91)	29.19 (.94)			
	6-week (*n* = 207)	26.82 (1.06)	27.80 (1.07)	−0.25(−3.50, 3.00)	0.88	0.20
	3 months (*n* = 202)	20.49 (.76)	21.43 (.75)	−0.29 (−3.14, 2.55)	0.84	0.11
PQ	Baseline (*n* = 410)	13.28 (.21)	13.41 (.21)			
	6-week (*n* = 366)	14.84 (.16)	14.44 (.15)	−0.25 (−0.88, 0.37)	0.43	0.30
	3 months (*n =* 357)	15.08 (.14)	14.95 (.13)	−0.53 (−1.18, 0.12)	0.11	0.38
Alabama Involvement	Baseline (*n* = 400)	34.92 (.62)	34.59 (.62)			
	6-week (*n =* 359)	33.83 (.66)	32.94 (.64)	0.56 (−2.65, 1.54)	0.60	0.13
	3 months (*n* = 352)	32.01 (.65)	31.81 (.61)	0.14 (−2.06, 2.34)	0.90	0.03
Alabama Supervision	Baseline (*n* = 408)	14.97 (.34)	14.82 (.34)			
	6-week (*n* = 364)	12.91 (.31)	13.54 (.29)	0.79 (−0.35, 1.92)	0.17	0.32
	3 months (*n* = 354)	12.43 (.25)	12.42 (.24)	0.15 (−0.91, 1.99)	0.79	0.06
Alabama Positive Parenting	Baseline (*n* = 407)	24.08 (.35)	24.63 (.35)			
	6-week (*n* = 362)	23.48 (.36)	23.60 (.34)	−0.43 (−1.59, 0.72)	0.46	−.17
	3 months (*n* = 352)	21.89 (.35)	22.30 (.33)	−.15 (−1.30, 0.99)	0.79	−.06
Alabama Discipline	Baseline (*n* = 406)	15.50 (.29)	14.74 (.28)			
	6-week (*n* = 364)	13.62 (.28)	13.53 (.26)	0.67 (−0.35, 1.69)	0.19	0.33
	3 months (*n* = 352)	13.00 (.27)	13.57 (.26)	1.32 (0.36, 2.29)	0.007	0.66
Alabama Punishment	Baseline (*n* = 410)	6.08 (.19)	6.34 (.19)			
	6-week (*n =* 365)	5.49 (.16)	5.55 (.16)	−0.20 (−0.75, 0.35)	0.47	−0.37
	3 months (*n* = 356)	5.48 (.14)	5.48 (.13)	−0.26 (−0.80, 0.27)	0.34	−0.27
PSC Attention Problems	Baseline (*n* = 374)	3.89 (.16)	6.45 (.16)			
	6-week (*n* = 322)	3.43 (.17)	5.78 (.17)	−0.21 (−0.78, 0.36)	0.46	−0.19
	3 months (*n* = 312)	3.15 (.16)	5.47 (.15)	−0.24 (−0.81, 0.33)	0.41	−0.22
PSC Internalizing	Baseline (*n* = 373)	3.22 (.11)	3.34 (.11)			
	6-week (*n* = 318)	2.83 (.12)	2.97 (.12)	0.01 (−0.39, 0.41)	0.96	0.01
	3 months (*n* = 305)	2.85 (.11)	3.01 (.11)	0.03 (−0.37, 0.42)	0.89	0.04
PSC Externalizing	Baseline (*n* = 372)	3.63 (.12)	3.64 (.12)			
	6-week (*n* = 322)	3.30(.11)	3.30 (.11)	−0.01 (−0.44, 0.42)	0.97	−0.01
	3 months (*n* = 311)	3.20 (.11)	3.37 (.10)	0.16 (−0.23, 0.56)	0.41	0.2

EUC, enhanced usual care; LS, least square; HSCL, Hopkins Symptom Checklist (depression subscale score range: 10–40; anxiety subscale score range: 15–60; higher scores indicate elevated anxiety or depression); WHODAS, WHO Disability Assessment Schedule (total score range: 0–48; higher scores indicate more severe impairment); PCL-5, Posttraumatic Stress Disorder Checklist (total score range: 0–80; higher scores indicate more severe PTSD severity); PSYCHLOPS, Psychological Outcomes Profiles (total score range: 0–20; higher scores indicate poorer outcome); PG-13, Prolonged Grief Disorder 13 (total score range: 11–57; higher scores indicate poorer outcome). PQ, Prodromal Questionnaire (total score range: 0–64; higher scores indicate poorer outcome). Alabama Parenting Questionnaire (Parental Involvement subscale score range: 10–50; Positive Parent subscale score range: 6–30; Supervision subscale score range 10–50; Discipline subscale score range 6–30; Punishment subscale score range 3–15; higher scores indicate elevated parental involvement, positive parenting, supervision, discipline, and punishment). Pediatric Symptom Checklist is child’s self-report (PSC; Attention Problems subscale score range: 0–10; Internalizing subscale score range: 0–10; Externalizing subscale score range: 0–14). Effect size was calculated by the difference in least square means between intervention and EUC from mixed model divided by the pooled standard deviation.

In terms of secondary outcomes, at 3 months, participants in the gPM+ condition had greater reductions in scores than EUC on the PSYCHLOPS (adjusted mean difference 0.88, 95% CI 0.07 to 1.69; *p* = .03), and the APQ discipline subscale (adjusted mean difference 1.54, 95% CI 1.03 to 2.05; *p* < .001). There were more participants in the gPM+ arm relative to EUC achieving a minimally important difference between baseline and 3-month follow-up for WHODAS (gPM+ 76.2%, EUC 66.1%); χ^2^ 6.67, *p* = 0.04). There were no significant differences at 3 months in changes of PTSD (adjusted mean difference −0.68, 95% CI −4.01 to 2.66; *p* = 0.69), disability (adjusted mean difference −1.21, 95% CI −2.80 to 0.37; *p* = .13), grief (adjusted mean difference −0.29, 95% CI −3.14 to 2.55; *p* = .84), prodromal symptoms (adjusted mean difference −0.53, 95% CI −1.18 to 0.12; *p* = .11), parental involvement (adjusted mean difference 0.14, 95% CI −2.06 to 2.34; *p* = .90), parental supervision (adjusted mean difference 0.15, 95% CI −0.91 to 1.99; *p* = .79) positive parenting (adjusted mean difference −0.15, 95% CI −1.30 to 0.99; *p* = .79), parental punishment (adjusted mean difference −0.26, 95% CI −0.80 to 0.27; *p* = .34), children’s attentional problems (adjusted mean difference −0.24, 95% CI −0.81 to 0.33; *p* = .41), children’s internalizing problems (adjusted mean difference 0.03, 95% CI −0.37 to 0.42; *p* = .89), or children’s externalizing problems (adjusted mean difference 0.16, 95% CI −0.23 to 0.56; *p* = .41).

The sensitivity analysis that focused only on participants that were retained at the 3-month follow-up did not change any of the results observed in linear mixed models analyses (S3 Table). Results of the covariate-adjusted analysis were consistent with those in the primary linear mixed model analysis; this indicated that degree of trauma exposure and ongoing stressors did not significantly impact results (S4 Table); the effect size for the depression outcome was higher after controlling for these factors relative to the initial analysis (0.90 versus 0.40). The analysis that focused only on participants with probable anxiety or depressive disorder indicated that the same results were observed as with the primary analyses; specifically, gPM+ resulted in greater reductions in depression, personally identified problems, and disciplinary parenting relative to EUC (S5 Table).

**Fig 2:**
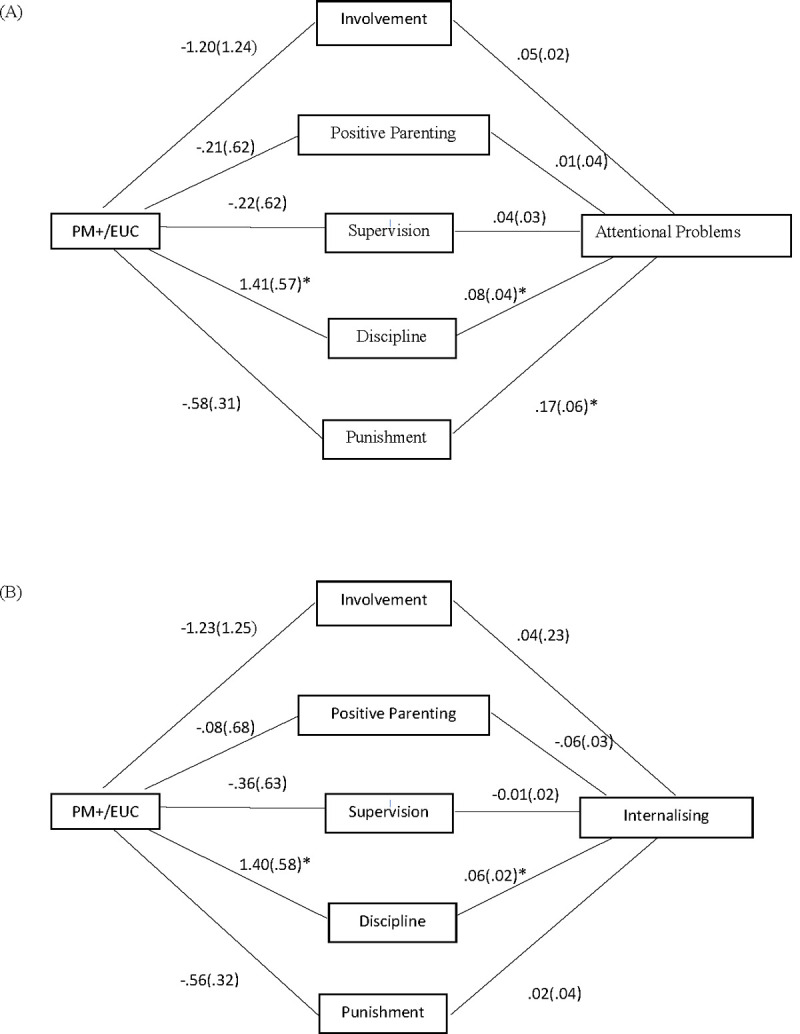
Path model of relationship between intervention, change in parenting style, and change in children’s mental health. Path model demonstrates that for refugees in gPM+, the more disciplinary parenting decreased (as measured by the APQ), there was a greater decrease in their children’s attentional (A) and internalizing (B) problems (as measured by the PSC). APQ, Alabama Parenting Questionnaire; EUC, enhanced usual care; gPM+, Group Problem Management Plus; PM+, Problem Management Plus; PSC, Pediatric Symptoms Checklist. Value are unstandardized coefficients (standard error). *Significant paths.

The mediation analysis indicated significant indirect paths between receiving gPM+ and greater reductions in the children’s reported attentional (β = 0.11, SE .07; 95% CI .003, .27) and internalizing (β = 0.08, SE .05; 95% CI .003, .19) problems were mediated through reductions in caregivers’ disciplinary (Figs 2 and S1). These patterns suggest that gPM+ was associated with improvements in children’s attentional and internalizing problems when there was an increase in consistent disciplinary behaviors in caregivers.

## Discussion

This trial tested the effectiveness of the WHO gPM+ intervention in Syrian refugees who were living in a secure refugee camp and who had fled the war in Syria in recent years and reported psychological distress. The major findings were that gPM+ significantly reduced depression and disability in refugees and also reduced disciplinary parenting behavior. The intervention did not demonstrate reductions in anxiety, PTSD, grief, prodromal symptoms, or child mental health problems.

The finding of reduced depression, disability, and personally identified problems is consistent with previous reports of PM+ [10,11] and gPM+ [12]. The demonstration that gPM+ can be effective in camp-based refugees confirms the useful brief mental health intervention in these settings. gPM+ relies on participants implementing strategies to engage in problem-solving, behavioral activation, and accessing social support; some of these activities may be limited in the camp context because of restricted movement within the camp, limited opportunities to gain employment, and constraints on making decisions that impact on one’s future. Most refugee camps around the world lack sufficient specialist mental health services, and so being able to implement programs such as gPM+ delivered by nonspecialists offers a scalable means to address common mental disorders of refugees in these settings. It is noteworthy that despite a brief training of only 8 days for the group facilitators in gPM+, the group sessions resulted in improved mental health; this amount of training is comparable with other evidence-based psychosocial programs [56] and is an important requisite for scalable interventions. Group-based programs may be more cost-effective than individually administered psychological interventions [57], and when they can be effectively delivered by nonspecialists, this further increases the likelihood that programs such as gPM+ could be implemented in refugee camps in poorly resourced countries [58]. Further, group interventions can be more acceptable in collectivist societies, including Syrian refugees [36].

Interestingly, and in contrast to prior trials of PM+ and gPM+ in community settings [10,12], the intervention did not reduce anxiety or PTSD levels relative to EUC. Moreover, the effect sizes observed in this study were generally lower than those observed in prior trials of gPM+ [12]. We also observed that there were no significant differences between treatment arms in terms of PTSD, disability, grief, prodromal symptoms, or childhood mental health outcomes levels. It is noteworthy that previous trials of PM+ or gPM+ have reported reductions in PTSD and disability [10,12]. The discrepancies between the current findings and those of other trials may be attributed to several factors. First, the post-migration challenges in the camp and insecurity of possibly being returned to Syria may have contributed to persistent mental health difficulties. In this context, it is worth noting that many refugees reported significant stressors in the camp, including poverty, fear for those who are in Syria, being separated from family, and loneliness. Further, when these factors were controlled for in the secondary analysis, we noted that the effect size for reduced depression in gPM+ was markedly larger than when they were not controlled for; this supports the conclusion that the ongoing stressors in the camp may have mitigated the potential gains of gPM+. Second, 61.5% of the participants met criteria for PTSD at entry to the study, and many had reported exposure to severe traumatic events; this severity of symptomatology and history of trauma exposure may reflect a challenge for brief transdiagnostic interventions with a population such as refugees recently exposed to war and experiencing high rates of PTSD living under harsh conditions of a refugee camp. Previous trials have not comprised war-affected refugee populations, and this trial may have recruited people with more severe psychopathology responses. Third, we note that the follow-up assessments occurred in the context of the COVID-19 pandemic when there were additional stressors in the camp, lockdowns, and fears of virus spread. This may have limited the gains in this trial relative to other trials. It is interesting that the interpretation that camp stressors may have limited the extent to which gPM+ reduced anxiety and other mental health outcomes does not apply to the same extent to depression. It is possible that the skills taught in gPM+ assisted refugees in managing depression-related problems such as social withdrawal and passivity, but problems associated with anxiety and traumatic stress were not so strongly targeted. Considering the prominence of trauma histories in this sample, it is possible that integrating a component into gPM+ that involves addressing trauma memories may provide stronger treatment effects. There is evidence that this form of treatment is effective in reducing posttraumatic stress in refugees [59].

A particularly novel finding was that gPM+ reduced maladaptive disciplinary parenting behavior in refugees. Harsh discipline may include attempts to control a child by verbal (shouting) or physical (hitting) abusive means. There is much evidence that harsh disciplinary parenting can lead to a range of child emotional and behavioral problems, including poor school performance [60,61]. Interestingly, gPM+ resulted in reduced harsh disciplinary behavior even though it is not an explicit parenting program. It can be speculated that the improvements in depressive mood, reduction in personal problems, and acquisition of problem management skills resulted in less maladaptive parenting behavior [62]. Although only an exploratory analysis was conducted with participants who completed the 3-month follow-up, we observed that gPM+ was associated with reduced attentional and internalizing problems in refugees’ children via the caregivers’ reduced disciplinary behavior. This accords with evidence from parenting programs that reducing maladaptive discipline can have positive impacts on children’s mental health [63,64]. This is an important finding because, although tentative at this stage, it suggests that brief behavioral programs aimed at refugees can have an indirect benefit on their children by reducing maladaptive parenting practices. This conclusion is supported by recent pilot research with parenting programs of Syrian refugees, which has found that it improved caregivers’ estimates of their children’s psychological well-being [17]. In this context, it is noteworthy that responses made during our cultural adaptation of gPM+ indicated a potential benefit in adding parental training; however, this component could not be accommodated in the current trial. Taken together, these qualitative and quantitative data suggest that future adaptations could usefully test gPM+ with parent training for caregivers [36].

Strengths of this study included random allocation, adherence to the treatment protocols, use of nonspecialist providers, extensive cultural adaptation of gPM+ for Syrian refugees, and excellent retention at follow-up. It is difficult to achieve the rigor of a randomized controlled trial in a secure refugee camp; however, this study adhered to the standards of controlled trials. Limitations included use of some measures that have not been culturally validated. Whereas the HSCL, WHODAS, and PCL have been validated in Arabic populations, other measures used in this trial were not culturally adapted for this population. This lack of proper adaptation may result in use of linguistically inappropriate idioms, failure to measure the intended constructs in Syrian refugees, and also the adapted measures may not possess optimal psychometric properties. We also note that provision of weekly group contact was not matched across conditions, and there was a preponderance of females in the study. Participation of males in psychological trials is much needed in global mental health; however, they are typically difficult to recruit into programs. Concerted efforts are needed in future research and implementation programs to recruit more men to psychosocial programs. We were also not able to obtain objective documentation regarding the services provided by health workers in the camp to EUC participants. Prescription of psychotropic medication is common practice, yet this information could not be detailed. Further, there is stigma regarding consulting a psychologist or mental health professional in this setting, and so there may be underreporting or poor attendance to these services; many refugees may consult physicians instead of mental health professionals. Although we retained 87.1% of the sample at follow-up, and secondary analyses indicated that this did not impact our findings, we recognize that the dropouts from gPM+ and the follow-up assessments are a limitation. Finally, we do not have data to validate the efficacy of the masking of assessors, such as by having assessors guess the participants’ allocated condition; this requirement is not in the CONSORT checklist, and we note that guessing the allocated treatment arm may be influenced by observation of the participant’s comments rather than reflecting efficacy of masking procedures [65]. Nonetheless, having this information may provide stronger evidence that masking was successfully achieved.

In terms of implications for clinical practice, the current findings suggest that gPM+ is an effective and scalable intervention that can be applied in refugee camps. The stressors faced by many refugees in refugee camp settings indicate that there is a need for scalable mental health interventions that can be delivered by lay providers in these contexts. Recognizing the lack of success in reducing PTSD and other secondary outcomes, the current findings suggest that different interventions may be indicated for these more complex clinical conditions. It is possible that the most effective framework to address more severe psychological conditions is a stepped care system, in which refugees who do not respond to gPM+ are offered a more intensive mental health program. In this context, it is worth noting that there are evidence-based trauma-focused therapies that can be applied to refugees with PTSD, which may be provided as a more specialist intervention for those who do not respond to gPM+. Alternately, people could be triaged in a way that those who meet criteria for PTSD may be referred for more intensive treatment instead of receiving gPM+. Further, the finding that gPM+ led to reductions in inconsistency disciplinary parenting behavior, and the observation that this was associated with reduced internalizing problems in their children, underscores the need for further research in how parenting can be targeted to improve the mental health of refugees’ children. In the context of elevated rates of mental health problems in refugee children [13], and the scarcity of effective, brief programs to improve children’s emotional and behavioral problems, it is possible that addressing the mental health of refugees and their parenting practices may provide a significant and cost-effective means to address this major global mental health issue.

In conclusion, considering the many refugees around the world in camps, the prevalence of mental disorders in these populations, and the scarcity of resources to provide effective mental health programs, the current findings indicate that gPM+ is an effective program to improve the mental health of refuges that can be scaled up in these settings. We note that the effect sizes were not large, and it is likely that stepped care services may be needed to address the more severe mental health conditions of refugees. The finding that gPM+ can indirectly benefit the psychological well-being of refugees’ children by improving parenting behavior opens up opportunities to assist children via brief programs that can improve refugees’ mental health.

## Supporting information

S1 Checklist CONSORT Checklist for Reporting Clinical Trials. (DOC)

S1 Text Clinical trial protocol. (DOCX)

S1 Table Participant characteristics of participants who were retained and lost at follow-up. (DOCX)

S2 Table Minimally important differences (MIDs) for primary and secondary outcomes. (DOCX)

S3 Table Summary statistics and results from mixed model analysis of primary and secondary outcomes for participants who completed 3-month assessment. (DOCX)

S4 Table Summary statistics and results from mixed model analysis of primary and secondary outcomes controlling for trauma exposure and post-migration living difficulties. (DOCX)

S5 Table Summary statistics and results from mixed model analysis of primary and secondary outcomes for participants with probable mental disorder (defined as mean item score of ≥2 on the Hopkins Symptom Checklist). (DOCX)

S1 Fig Path model of relationship between gPM+/EUC, change in parenting style, and change in children’s externalizing problems. Path model demonstrates that there was a significant relationship between gPM+ and reductions in disciplinary parenting (as measured by the APQ) but no association between parenting style and externalizing problems (as measured by the PSC). Value are unstandardized coefficients (standard error). *Significant paths. APQ, Alabama Parenting Questionnaire; EUC, enhanced usual care; gPM+, Group Problem Management Plus; PSC, Pediatric Symptoms Checklist.
(DOCX)

## Abbreviations

APQ: Alabama Parenting Questionnaire
EUC: enhanced usual care
gPM+: Group Problem Management Plus
HSCL: Hopkins Symptom Checklist
IMC: International Medical Corps
K10: Kessler Psychological Distress Scale
LMICs: low- and middle-income countries
PGD: prolonged grief disorder
PQ-B: Prodromal Questionnaire-16
PSC: Pediatric Symptoms Checklist
PTSD: posttraumatic stress disorder
RCT: randomized controlled trial
WHODAS: WHO Disability Assessment Schedule
