# Supplementary material for: Effectiveness of a brief group behavioral intervention for common mental disorders in Syrian refugees in Jordan: A randomized controlled trial
Source: PLoS Med. 2022 Mar 17;19(3):e1003949. doi: 10.1371/journal.pmed.1003949 (PMC8929659; doi:10.1371/journal.pmed.1003949)
Supplement: S1 Text — (DOCX) [file pmed.1003949.s002.docx]

**RESEARCH PROTOCOL:**

**Improving Mental Health of Syrian Refugees in Jordan**

**(February 2018)**

**University of New South Wales Human Research Ethics Committee**

**PROTOCOL TITLE ‘Improving Mental Health of Syrian Refugees in Jordan’**

| **Short title** | **Improving Mental Health in Syrian Refugees** |
| --- | --- |
| **Version** | **1** |
| **Date** | **June 6, 2017** |
| **Coordinating investigator/project leader** | ***Professor Richard Bryant*** |
| **Principal investigator(s)** | ***Professor Richard Bryant***  ***School of Psychology, UNSW*** |
| **Sponsor** | ***UNSW*** |
|  |  |
| **Subsidising party** | ***NHMRC*** |
|  |  |
| **Laboratory sites <*if applicable*>** | ***Not applicable*** |
|  |  |
| **Pharmacy <*if applicable*>** | ***Not applicable*** |
|  |  |

**PROTOCOL SIGNATURE SHEET**

| **Name** | **Signature** | **Date** |
| --- | --- | --- |
| ***<For non-commercial research,>***  **Head of Department:**  ***Prof Simon Killcross*** |  |  |
| **[Coordinating Investigator/Project leader/Principal Investigator]:**  **Prof Richard Bryant** |  |  |

**TABLE OF CONTENTS**

1. INTRODUCTION AND RATIONALE 10

2. OBJECTIVES 13

3. STUDY DESIGN 14

4. STUDY POPULATION 17

4.1 Population (base) 17

4.2 Inclusion criteria 17

4.3 Exclusion criteria 17

4.4 Sample size calculation 17

5. TREATMENT OF SUBJECTS 19

5.1 Investigational product/treatment 19

5.2 Use of co-intervention (if applicable) 20

5.3 Escape medication (if applicable) 21

6. METHODS 22

6.1 Study parameters/endpoints 22

6.1.1 Main study parameter/endpoint 22

6.1.2 Secondary study parameters/endpoints (if applicable) 22

6.1.3 Other study parameters (if applicable) 22

6.2 Randomisation, blinding and treatment allocation 23

6.3 Study procedures 23

6.4 Withdrawal of individual subjects 33

6.4.1 Specific criteria for withdrawal (if applicable) 34

6.5 Replacement of individual subjects after withdrawal 34

6.6 Follow-up of subjects withdrawn from treatment 34

6.7 Premature termination of the study 34

7. SAFETY REPORTING 35

7.1 Temporary halt for reasons of subject safety 35

7.2 AEs, SAEs and SUSARs 35

7.2.1 Adverse events (AEs) 35

7.2.2 Serious adverse events (SAEs) 35

7.3 Follow-up of adverse events 36

7.4 Safety Committee 36

8. STATISTICAL ANALYSIS 38

8.1 Primary study parameter(s) 38

8.2 Secondary study parameter(s) 39

8.3 Other study parameters 39

8.4 Interim analysis (if applicable) 40

9. ETHICAL CONSIDERATIONS 41

9.1 Regulation statement 41

9.2 Recruitment and consent 41

9.3 Objection by minors or incapacitated subjects (if applicable) 42

9.4 Benefits and risks assessment, group relatedness 42

9.5 Compensation for injury 42

9.6 Incentives (if applicable) 43

10. ADMINISTRATIVE ASPECTS, MONITORING AND PUBLICATION 44

10.1 Handling and storage of data and documents 44

10.2 Monitoring and Quality Assurance 44

10.3 Amendments 45

10.4 Annual progress report 45

10.5 Temporary halt and (prematurely) end of study report 45

10.6 Public disclosure and publication policy 46

11. REFERENCES 47

**LIST OF ABBREVIATIONS AND RELEVANT DEFINITIONS**

| **ABR** | **ABR form, General Assessment and Registration form, is the application form that is required for submission to the accredited Ethics Committee (In Dutch, ABR = Algemene Beoordeling en Registratie)** |
| --- | --- |
| **AE** | **Adverse Event** |
| **AR** | **Adverse Reaction** |
| **CSRI** | **Client Service Receipt Inventory** |
| **DSM** | **Diagnostic and Statistical Manual of Mental Disorders** |
| **EU** | **European Union** |
| **GCP** | **Good Clinical Practice** |
| **HSCL** | **Hopkins Symptoms Checklist** |
| **IC** | **Informed Consent** |
| **K-10** | **Kessler Psychological Distress Scale (ten item version)** |
| **LEC** | **Life Events Checklist** |
| **METC** | **Medical research ethics committee (MREC); in Dutch: medisch ethische toetsing commissie (METC)** |
| **PCL** | **PTSD Checklist** |
| **PM+** | **Problem Management Plus** |
| **PMLD** | **Post-Migration Living Difficulties** |
| **PSYCHLOPS** | **Psychological Outcome Profiles instrument** |
| **PTSD** | **Posttraumatic Stress Disorder** |
| **RCT** | **Randomized Controlled Trial** |
| **SB** | **Safety Board** |
| **Sponsor** | **The sponsor is the party that commissions the organisation or performance of the research, for example a pharmaceutical**  **company, academic hospital, scientific organisation or investigator. A party that provides funding for a study but does not commission it is not regarded as the sponsor, but referred to as a subsidising party.** |
| **STRENGTHS** | **Syrian REfuGees MeNTal HealTH Care Systems** |
| **WHODAS** | **WHO Disability Assessment Schedule** |
| **WHO** | **World Health Organization** |
| **WMO** | **Medical Research Involving Human Subjects Act (in Dutch: Wet Medisch-wetenschappelijk Onderzoek met Mensen** |

**SUMMARY**

**Rationale:** The current refugee crisis across the Middle East and Europe has large effects on individual refugees’ psychological wellbeing, as well as on the healthcare systems of countries housing refugees. The WHO have developed Problem Management Plus (PM+), a brief (five-sessions), low-intensity psychological intervention, delivered by paraprofessionals, that addresses common mental disorders in people in communities affected by adversity.

**Objective**: The main objective is to evaluate feasibility, acceptability, effectiveness and cost-effectiveness of the culturally adapted PM+ intervention for Syrian refugees in Jordan. The main hypothesis is that PM+ will decrease psychological distress as compared to treatment as usual only. The objectives per study phase are: To obtain estimates of drop-out rates to inform a full-scale, definitive RCT (Study Phase 2); to understand the perceptions of key stakeholders with regards to PM+ intervention (Study Phases 3 and 5); and to test effectiveness and cost-effectiveness of the PM+ intervention (Study Phase 4). The objective of Study Phase 1 is to obtain translation and (cultural) adaptation of PM+ for Syrian refugees in The Netherlands. This Study Phase is discussed in a separate protocol.

**Study design:** Study Phase 2: exploratory, single-blind randomized controlled trial (RCT), Study Phase 3 and 5: qualitative study, Study Phase 4: definitive single-blind RCT.

**Study population:** Adult female Syrian refugees (above 18yrs) who have a child between the ages of 10-16 years residing in Amman, with self-reported functional impairment (WHODAS 2.0 >16) and elevated psychological distress (K10 >15.9).

**Intervention (if applicable)**: Participants will be randomised to receive five sessions of Problem Management Plus (PM+), an evidence-based psychological intervention, or treatment as usual (TAU). PM+ is an evidence-based, low-intensity group intervention and will be delivered by trained providers in Jordan. The control group will receive TAU only.

**Main study parameters/endpoints:** The main study parameter will be the decrease in psychological distress from baseline to three-month follow-up, measured through the Hopkins Symptoms Checklist (HSCL-25), a self-report measure for symptoms of psychological distress. We expect a difference of Cohen’s *d* effect size of .4 between the PM+ group and controls. Secondary parameters include functional impairment (WHODAS 2.0), posttraumatic stress reactions (PCL-5), self-identified problems (PSYCHLOPS), and cost of care (CSRI schedule). Additionally, mental health of one child of each participant will be assessed using the Strengths and Difficulties Questionnaire (SDQ),

**Nature and extent of the burden and risks associated with participation, benefit and group relatedness:** Interested participants will be invited for a total of four assessment interviews over a period of one year. The interviews include questionnaires on daily functioning and psychological complaints, adverse experiences, daily hassles and cost of care. The interview will take approximately 1.0hrs. Participants in the treatment group will receive five sessions PM+.

# INTRODUCTION AND RATIONALE

Recent crises in the Middle East, most notably in Syria, have resulted in an unprecedented increase in the number of refugees seeking asylum in neighbouring countries as well as in Europe. In 2015, over 1 million refugees have been registered entering Europe through the Mediterranean Sea (UNHCR, December 31 2015), and 4.8 million have fled to Syria’s neighbouring countries. Reports state that over 50% of Syrian refugees are children, in many cases unaccompanied by their family (UNHCR, 2016; UNICEF, 2016).

Refugees may have been exposed to multiple war stressors such including sexual violence and destruction of their homes and livelihoods, and they have often undertaken a risky and stressful flight leaving their homes for an unknown future. Studies show that refugees are at considerable risk to develop common mental disorders, including depression, anxiety, posttraumatic stress disorder (PTSD) and related somatic health symptoms (Steel et al., 2009).

Recent World Health Organization projections suggest that approximately 15-20% of Syrian refugees will have/deal with/develop some type of mental health issue (Hassan et al., 2015). Children refugees are especially at high risk for developing emotional problems, with a recent study in Syrian refugee children in Turkey reporting that nearly half of them show clinically significant levels of anxiety and withdrawal (Cartwright, El-Khani, Subryan, & Calam, 2015).

The refugee crisis imposes highly challenging demands on health systems in Europe and the Middle East. Given it adjacent position to Syria, Jordan hosts over 600,000 Syrian refugees. This has resulted in a sudden steep increase in numbers of individuals with mental health needs, and Jordan’s current health infrastructure is challenged to meet this need. In other countries in the Middle East and North Africa (MENA), such as Turkey and Lebanon, the mental health services required to meet the demands of millions of refugees in need are similarly inadequate and their health systems are overburdened to meet even basic survival needs and more chronic health problems (Gornall, 2015). The provision of services to address the psychological conditions of millions of refugees is conducted by non-government organisations (NGOs) and coordinated by international organizations, including the International Federation of Red Cross, United Nations High Commissioner for Refugees (UNHCR), International Medical Corps (IMC) and many others, including the Noor Al Hussein Foundation in Jordan.

The demand for scarce mental health services exceeds their availability in countries surrounding Syria, and there are also numerous barriers to the delivery and uptake of available services. Barriers to the delivery and uptake of mental health interventions for refugees include a lack of financial resources to pay for lengthy treatment programs, and limited capacity of mental health care specialists to deliver specialized services. In addition, the length and complexity of specialized treatments preclude simple access for refugees who are often unable to attend regular treatments over extended periods of time and to travel to attend sessions. Moreover, mental health programs usually focus on single psychiatric disorders (such as posttraumatic stress disorder; PTSD), whereas many refugees suffer multiple psychological problems that extend beyond single diagnostic boundaries (Thabet, Abed, & Vostanis, 2004). Many refugees suffer from general psychological distress that does not need specialized mental health care interventions, but brief psychological interventions could be helpful to prevent more serious disorders. Finally, knowledge within refugee populations about mental health care supply is limited and stigma concerning mental health is prevalent in refugee populations and so many are reluctant to seek mental health care through formal services.

The World Health Organization has developed the low-intensity Problem Management Plus (PM+) programmes, a new generation of shorter, less expensive and trans-diagnostic (i.e., not condition-specific) programs to reduce common mental health symptoms and improve psychosocial functioning. It is based on the WHO treatment guidelines for conditions related to stress (WHO, 2013). PM+ is a 5-sessions *intervention* (Dawson et al., 2015) that reduces *symptoms of depression, anxiety, PTSD, and related conditions*, is delivered by *trained non-specialized workers or lay people*, and is available in individual and group delivery formats for both children and adults. It comprises evidence-based techniques: of (a) problem solving, (b) stress management, (c) behavioural activation, and (d) accessing social support. PM+ has been successfully implemented in Kenya and Pakistan (Bryant, Dawson, Schafer, Sijbrandij, & van Ommeren, 2016; Rahman, Riaz, & Dawson, n.d.; Atif Rahman, Hamdani, Awan, Bryant, Dawson, Khan, Mukhtar-ul-Haq Azeemi, et al., 2016).

In a randomized controlled trial (RCT) in Kenya 410 women exposed to gender-based violence were randomized to receive PM+ or enhanced treatment as usual (TAU). Local volunteer health workers with no mental health experience were trained in PM+, and delivered 5 sessions of PM+ to each participant. Relative to TAU at a three-month follow-up assessment, PM+ resulted in greater reductions in anxiety and depression, posttraumatic stress, and functional disability (Bryant et al., 2016). This RCT also found fewer days of job absence in women who received PM+ than control participants. Similarly, a controlled trial of 344 people in Pakistan affected by terrorism and war found that those who received five sessions of PM+ had greater reductions in anxiety, depression, functional disability, and posttraumatic stress than those who received an enhanced treatment as usual (Atif Rahman, Hamdani, Awan, Bryant, Dawson, Khan, Mukhtar-ul-Haq Azeemi, et al., 2016). As a result, the WHO has adopted PM+ as the key low-intensity mental health intervention approach to be globally implemented within primary and community health care to populations affected by adversity.

In the **STRENGTHS** (**S**yrian **RE**fu**G**ees Me**NT**al Heal**TH** Care **S**ystems) consortium, a series of studies will evaluate the (cost)effectiveness of PM+ in Syrian refugees. In this trial, group-based PM+ will be evaluated in Jordan. Two very similar study protocols for research to the effectiveness of PM+ in Kenya and Pakistan have previously been approved by the Ethics Review Committee of the WHO. The principal investigator of this study, Professor Richard Bryant, led the study in Kenya and was a co-investigator in the Pakistan trial.

# OBJECTIVES

Primary Objective:

To evaluate feasibility, acceptability, effectiveness and cost-effectiveness of the culturally adapted PM+ intervention for Syrian refugees in Jordan.

Secondary Objective(s):

1. To translate and (culturally) adapt PM+ for use among Syrian refugees in Jordan (*Study Phase 1, described in a separate protocol, see HREAP)*
2. To conduct a pilot study to obtain estimates of drop-out rates to inform a full-scale, definitive randomized controlled trial (*Study Phase 2*)
3. To understand the perceptions of key stakeholders with regards to PM+ intervention (*Study Phases 2 and 4*)
4. To test effectiveness and cost-effectiveness of the PM+ intervention (*Study Phase 3*)

# STUDY DESIGN

This research entails qualitative study (Study Phases 2 and 4) and single-blind randomized controlled trials (Study Phase 1 and 3). We will conduct an exploratory randomized controlled trial (RCT) and a definitive RCT to test the (cost-)effectiveness of the culturally adapted Problem Management Plus (PM+) intervention (WHO, 2016). These trials will be informed and evaluated by quantitative and qualitative methods.

| Box 1: Study Phases for STRENGTHS Pragmatic Trials |
| --- |
| Study Phase 1: Translation and (cultural) adaptation PM+ (discussed in separate protocol)  Study Phase 2: Exploratory randomized controlled trial  Study Phase 3: Qualitative evaluation pilot study  Study Phase 4: Definitive randomized controlled trial  Study Phase 5: Process evaluation |

*Study Phase 1*

Study Phase 1 is described in a separate protocol.

*Study Phase 2*

The exploratory RCT will inform us about the feasibility, safety and delivery of the intervention; and that will identify issues around its training, supervision and outcome measures. STRENGTHS’s research strategy is informed by the UK Medical Research Council framework for the development of complex interventions, which recognizes iterations of: a) Intervention Development; b) Feasibility and Piloting (trial 1a); c) Evaluation; and d) Implementation (trial 1b) (Craig et al., 2013). ‘Intervention Development’ is part of the cultural adaptation process in Work Package 3 of STRENGTHS and has already been conducted by the Danish Red Cross. This key framework for development of interventions recommends exploratory and randomized pilot studies prior to large scale trials to address uncertainties such as problems of acceptability, compliance, delivery of the intervention, recruitment and retention, and smaller than expected effect sizes (Craig et al., 2013). The exploratory RCT (Phase 1), which likely is underpowered to show efficacy, will provide the information necessary (drop-out rates etc.) to inform the definitive RCT (Phase 3).

*Study Phase 3*

In Study Phase 3, we will evaluate barriers and facilitators among key stakeholders for large-scale implementation of PM+ through key informant interviews (Phase 2 and 4 participants, peer-refugees, mental health professionals and policy makers). Interviews will explore barriers and facilitators to treatment engagement and adherence, as well as the opportunities for scaling up the implementation of the intervention within the existing healthcare system in Jordan. This information will support study design of a definitive RCT, providing qualitative data about PM+ implementation, complementing the quantitative measures of Study Phase 2 (Bolton, Tol, & Bass, 2009).

*Study Phase 4*

In Study Phase 4, we will conduct a definitive RCT to evaluate effectiveness and cost-effectiveness of PM+ in 346 study participants.

*Study Phase 5*

In Study Phase 5, we will again evaluate barriers and facilitators among key stakeholders. Interviews will explore barriers and facilitators to treatment engagement and adherence, as well as the opportunities for scaling up the implementation of the intervention within the existing healthcare system in Jordan. It will inform partners in STRENGTHS for the synthesis and dissemination of PM+ for Syrian refugees.^[[1]](#footnote-1)^

| *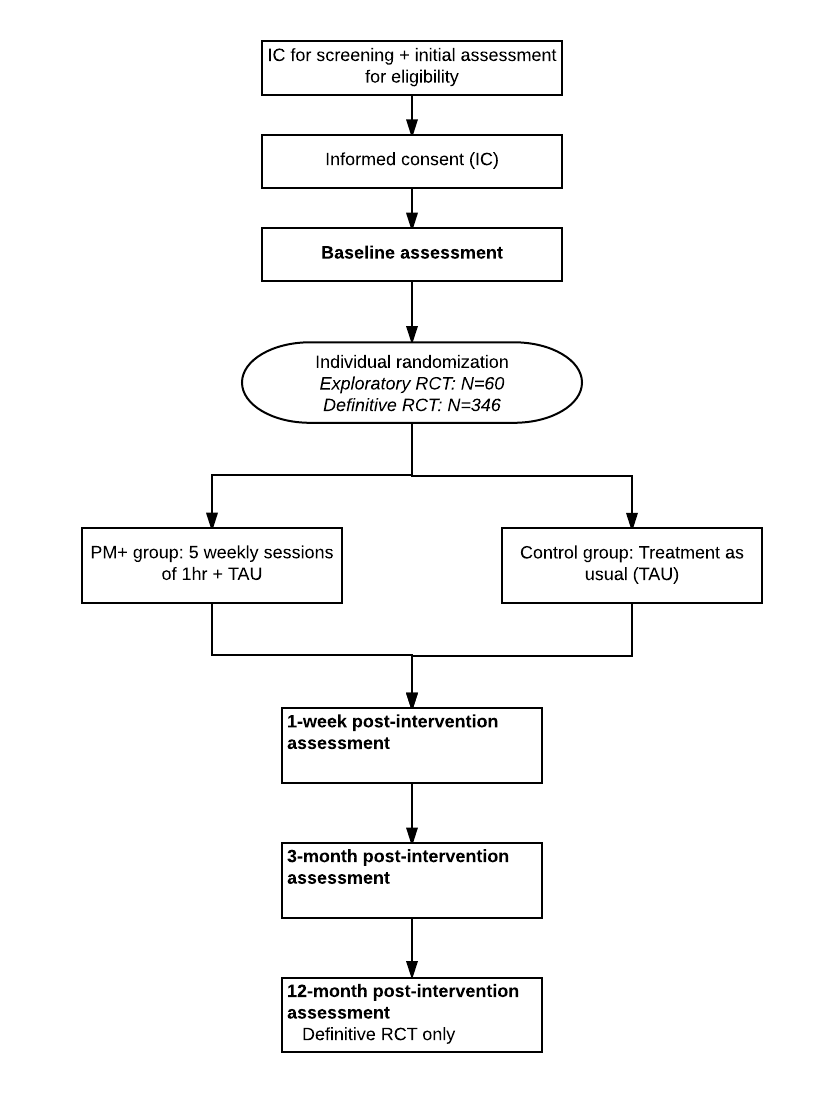* |
| --- |
| *Figure 1.* Flowchart for Exploratory and Definitive RCTs |

# STUDY POPULATION

## Population (base)

Participants will be adult female Syrian refugees residing in Jordan. The total influx of Syrian refugees is over 650,000. The current study will be carried out in [a community setting in Amman. The PM+ intervention will be implemented by the Noor Al Hussein Foundation, a non-government organisation providing psychosocial and medical services in Jordan.

## Inclusion criteria

In order to be eligible to participate in this study, a subject must meet all of the following criteria:

- 18 years or above
- Syrian refugee status
- Have a child residing in the household aged 10-16 years
- Arabic-speaking
- Elevated levels of psychological distress (K10 >15.9) and reduced psychosocial functioning (WHODAS 2.0 >16)

## Exclusion criteria

A potential participant who meets any of the following criteria will be excluded from participation in this study:

- Acute medical conditions
- Imminent suicide risk or with expressed acute needs/protection risks (e.g., a woman who expresses that she is at acute risk of being assaulted or killed)
- Severe mental disorder (psychotic disorders, substance-dependence)
- Severe cognitive impairment (e.g., severe intellectual disability or dementia)

## Sample size calculation

*Statistical power and sample size*

. A total number of 346 participants will be included. Based on previous studies with PM+ carried out in Peshawar, Pakistan (Rahman et al., 2016), and Nairobi Kenya (Bryant et al., 2016), we aim for a conservatively estimated small to medium Cohen’s *d* effect size of 0.4 in the PM+ group at 3 months follow-up (the primary outcome timepoint). Power calculations suggest a minimum sample size of 133 participants per group (power = 0.90, *a* = 0.05, two-sided). Taking into account an expected 35% attrition at 3 months follow-up, we aimed to include a total number of 410 participants (210 in the gPM+ group and 210 in the care-as-usual control group).

# TREATMENT OF PARTICIPANTS

## Investigational product/treatment

*The PM+ intervention program*

PM+ is a new, brief, psychological intervention program based on cognitive behavioural therapy (CBT) techniques that are empirically supported and formally recommended by the WHO (Dua et al., 2011; Tol, Barbui, & van Ommeren, 2013; WHO, 2010b, 2013). The full protocol was developed by WHO and University of New South Wales, Australia. The manual involves the following empirically supported elements: problem solving plus stress management, behavioural activation, facing fears, and accessing social support. These elements have been recommended in recent WHO guidelines (Dua et al., 2011; Tol et al., 2013). Figure 2 shows a brief outline of the five sessions.

PM+ has four core features. It is:

1. Brief (five sessions);

2. Delivered by para-professionals;

3. Transdiagnostic, addressing depression, anxiety, PTSD, stress and problems as defined by people themselves, and

4. Designed for people in low-income country communities affected by adversity (e.g., armed conflict)

There is currently no programme that addresses all these features.

PM+ providers will be female providers with a background in health care, social work or community care, and who will receive two weeks of training. PM+ trained psychologists employed by Noor Al Hussein will be responsible for supervising the peer-refugee PM+ providers.^[[2]](#footnote-2)^ Protocol adherence will be ensured by the supervisors and two-weekly supervisions of the PM+ peer-refugee counsellors (Murray et al., 2011). A checklist will be developed to code a random sample of supervisor checked sessions on treatment fidelity.

|  |
| --- |
| *Figure 2.* Outline of Five PM+ Sessions (figure from PM+ Manual (WHO, 2016)) |

*The control condition*

The comparison group will receive treatment as usual (TAU).

## Use of co-intervention (if applicable)

Participants are allowed to use other medication or (psychological) treatments; participants in both groups (PM+ as well as treatment as usual) will continue to receive routine care on an individual basis.

## Escape medication (if applicable)

Not applicable.

# METHODS

## Study parameters/endpoints

The hypothesis to be tested is that PM+ (five weekly sessions) will decrease psychological distress as compared to treatment as usual only.

### Main study parameter/endpoint

The main study parameter will be the decrease in psychological distress from baseline to three-month follow-up, measured through the Hopkins Symptoms Checklist (HSCL-25). A description of the measure can be found under ‘Study Procedures’. We expect a between-group Cohen’s *d* effect size difference of .4, based on prior research on PM+ in Pakistan and Kenya (Bryant et al., 2016; Rahman, Hamdani, Awan, Bryant, Dawson, Khan, Mukhtar-ul-Haq Azeemi, et al., 2016). A subsequent follow-up assessment will be conducted at 12 months but this is a secondary outcome.

### Secondary study parameters/endpoints (if applicable)

The measurement instruments are described under ‘Study procedures’.

1. Level of functional impairment (WHODAS 2.0)
2. Severity of posttraumatic stress reactions (PCL-5)
3. Self-identified problems (PSYCHLOPS)

4. Number of adverse life events (LEC)

5. Post-Migration Living Difficulties (PMLD)

6. Cost of care (CSRI schedule)

7. Prolonged grief-13 (PG-13)

8. Prodromal Questionnaire-16 (PQ-B)

9. Alabama Parenting Questionnaire-42 (APQ)

10. Pediatric Symptoms Checklist (PSC)

### Other study parameters (if applicable)

The measurement instruments are described under ‘Study procedures’.

1. Demographic data (WHODAS 2.0)

2. Treatment fidelity (checklists)

3. Other indicators on intervention delivery: implementation process, adaptation, reach, dose, quality

## Randomisation, blinding and treatment allocation

The two trials (pilot and RCT) are single-blind randomized controlled trials (i.e., outcome assessors are blind to treatment allocation).

Randomization will be carried out by an independent researcher not involved in intervention delivery, clinical supervision, independent assessment or other aspects of the day-to-day running of the study. Randomization will be performed using computerized software on a 1:1 basis. If randomized into the PM+ condition, participants will be allocated by the independent researcher who performed the randomization. The first session will aim to schedule within a few days and not longer than two weeks after the pre-intervention assessment.

## Study procedures

**Study Phase 2: Exploratory randomized controlled trial (RCT) to evaluate feasibility of administering the culturally adapted PM+ for adult Syrian refugees in Jordan.**

The study will be carried out in Amman. The study will be overseen by Noor Al Hussein. Eligible participants will be adult (18 years or above) female Syrian refugees with a child between the ages of 10-16 years without acute medical conditions (see inclusion and exclusion criteria in paragraph 2.2 and 2.3). They will first be orally informed about the project by a staff member of Noor Al Hussein and will be asked whether they agree to hear about the research. Only if permission is given, a research assistant will meet with the eligible patient and will ask informed consent for screening. Participants will be free to decline to participate or withdraw at any time without affecting their routine care.

Participants will include adult Syrian refugees who: a) score above 15.9 on a 10-item screening questionnaire for common mental disorders (Kessler Psychological Distress Scale; K-10) (Kessler et al., 2002), and b) score above 16 on a screening questionnaire for functional impairments (WHO Disability Assessment Schedule 2.0; WHODAS 2.0) (WHO, 2010a). These instruments are administered by a research assistant and are described below (‘Measurement instruments’).

Individuals who meet the exclusion criteria as described in paragraph 2.3 will be excluded and referred for appropriate treatment and support. This refers to individuals with imminent suicide risk or with expressed acute needs/protection risks (for example, a woman who expresses that she is at acute risk of being assaulted or killed). Suicidal ideation will be explored through the PM+ ‘assessment of thoughts of suicide’ tool (WHO, 2016, pp. 86). In addition, we will also exclude individuals with severe mental disorder (psychotic disorders, substance-dependence) or severe cognitive impairment (eg., severe intellectual disability or dementia). Mental, neurological or substance use disorders will be screened with the ‘impairments possibly due to severe mental, neurological or substance use disorders’ tool of the PM+ intervention manual (WHO, 2016, pp. 87). Individuals meeting the exclusion criteria will be referred to specialist support in Noor Al Hussein or to the International Medical Corps, who provide comprehensive services in Ammans. If the patients agree to be referred, the assessment results will be provided to the treating mental health professional with permission of the patient and the research team will ensure that an appointment is made with a mental health professional.

If participants are not selected for the trial because they score below the cut-off scores for the K-10 or the WHODAS 2.0, they will be provided feedback on their test outcomes and will be explained why they are not eligible for the study (see pp. 88-89 of the PM+ intervention manual) (WHO, 2016).

The informed consent process entails a two-step procedure; 1. Informed consent for screening, and 2. Informed consent for taking part in the PM+ trial.

1. Participants will first be orally informed about the project by staff of Noor Al Hussein, and will be asked whether they agree that a member of the research team will provide them with further information about the study. Only if permission is given a research assistant will informed consent for study participation be requested. Participants will be free to decline to participate or withdraw at any time. Respondents who decide to participate will be asked to complete a written consent form. For participants who are illiterate, witnessed oral consent and a thumb print in lieu of a signature will be sufficient. The witness will be a member of the research staff and who is willing to act as the witness.

Following informed consent for screening, demographic characteristics will be recorded by the research assistant and participants will be invited to complete the K-10, the WHODAS 2.0, and the PM+ manual suicide tool. No identifiable information will be recorded.

1. If participants meet the eligibility criteria (K-10 >15.9 and WHODAS 2.0 >16), they will be given oral and written information about participating in the RCT by the research assistant. At least 24 hours after, a research assistant will ask informed consent to participate in the trial (see Screening Consent Form).

Following informed consent for participating in the trial, the K10, the WHODAS 2.0, the Psychological Outcome Profiles instrument (PSYCHLOPS), the Life Events Checklist (LEC), the PTSD Checklist for DSM-5 (PCL-5), the Post-Migration Living Difficulties (PMLD), and the Client Service Receipt Inventory (CSRI) will be administered. Also, the Strengths and Difficulties Questionnaire will be completed by the child.

After the assessment, participants will be randomized by an independent research assistant, not involved in the assessments, in either the PM+ intervention (*n*=30) or the treatment as usual (TAU) control condition (*n*=30). Randomization will be performed using computerized software on a 1:1 basis. Since this is a small exploratory RCT (to inform a definitive RCT) that does not aim to detect statistically significant differences in effectiveness, no power calculations have been carried out. We intend to deliver the intervention to 30 participants in each arm, allowing us to test the feasibility and acceptability of the intervention in the proposed setting, as well as drop-out rates for a future definitive trial. Randomization will be carried out by an independent researcher not involved in intervention delivery, clinical supervision, independent assessment or other aspects of the day-to-day running of the study. If randomized into the PM+ condition, participants will be allocated to an intervention therapist by the independent researcher who performed the randomization. The first session will be scheduled within a few days and not longer than one week after the pre-intervention assessment.

The post-intervention assessment (WHODAS 2.0, K10, PSYCHLOPS, PMLD, CSRI schedule, and additional questions on perceived access to health services) will be scheduled seven weeks after the pre-intervention assessment (i.e., one week after the fifth PM+ session, see Figure 1). The follow-up assessment will be conducted three months after the fifth PM+ session. All instruments of the post-intervention assessment, including the LEC, will be administered again at follow-up.

In case participants do not attend a scheduled assessment, they are called a maximum of five times (on different days) for scheduling a new appointment.

*Assessors*

All instruments will be administered by trained research staff blind to the allocation status of the participants. All assessors will receive a five day training in administering the instruments, in general interview techniques, and in responding to participant distress, including, as mentioned, psychological first aid. The training will be delivered by the research team who are attuned to potential mental health difficulties interviewers may be facing as a result of the adversity. If indicated, psychologists will conduct full assessments where there are questions about the capacity of an individual to carry out their role effectively. Ongoing monitoring of assessors’ capacity to practice will be conducted through regular supervision of assessors by the main investigator. This oversight will help ensure that any potential concerns about the capacity of assessors to carry out their roles is picked up and responded to.

*Measurement instruments*

The eleven measures that will be used for the baseline and post-intervention assessments are depicted in Table 1.

| Table 1. *Overview of Measures* | | | |
| --- | --- | --- | --- |
|  | Measures | | |
| Concept | Baseline | PM+ sessions | Post-intervention |
| Functioning | WHODAS 2.0 ^a^ |  | WHODAS 2.0 ^a^ |
| Distress | K10 ^b^ |  | N/A |
|  | HSCL-25 ^c^ |  | HSCL ^c^ |
| Posttraumatic stress  reactions | PCL-5 |  | PCL-5 |
| Self-identified  problems | PSYCHLOPS |  | PSYCHLOPS |
| Children’s mental health | SDQ |  | SDQ |
| Adverse life events | LEC |  | LEC ^d^ |
| Post-migration  stressors | PMLD |  | PMLD |
| Cost of care | CSRI schedule |  | CSRI schedule |
| Prolonged grief | PG-13 |  | PG-13 |
| Prodromal psychosis | PQ-B |  | PQ-B |
| Parenting behaviour | APQ |  | APQ |
| Child’s mental health | PSC |  | PSC |
| Treatment fidelity |  | Fidelity checklists |  |
| ^a^ Screener and secondary outcome measure  ^b^ Screener only  ^c^ Primary outcome measure  ^d^ Time period since baseline, only measured at 12-month follow-up | | | |

**Screeners**

*WHODAS: socio-demographic information and disability*

Data on socio-demographic information (sex, age, education and marital status) will be collected through questions A1-A5 of the WHO Disability Assessment Schedule 2.0 (WHODAS 2.0) (WHO, 2010c), which will be administered first.

The WHODAS is a generic assessment instrument assessing health and disability. It is used across all diseases, including mental neurological and substance use disorders. It is simple to administer and applicable across cultures and can be used in all adult populations. The WHODAS covers six domains (cognition, mobility, self-care, getting along, life activities, participation). It assesses difficulties people have due to their illness across these domains during the last 30 days. Difficulties are scored on a five-point Likert scale ranging from 0 (*none*) to 4 (*extreme*), before summation (range 0-48). Higher scores indicate worse functional impairment. We will use the 12-item interviewer administered version, which has been validated in different cultural contexts (WHO, 2010a).

*K10: psychological distress*

Psychological distress will be measured through the Kessler-10 Psychological Distress Scale (Kessler et al., 2002). Ten items related to distress are rated on a five-point Likert scale, and an additional four questions are asked to assess the degree of disability. The sum of the ten items gives a total score ranging from 10 to 50. The four additional questions do not contribute to the total score. The Arabic version of the K10 is validated by the Transcultural Mental Health Centre in NSW, Australia (Sulaiman-Hill & Thompson, 2010).

In a study among Kurdish and Afghan (former) refugees and asylum seekers in New Zealand and Australia, the following cut-off scores were used: 10-15.9 (*low risk of psychological distress*), 16-21.9 (*moderate levels of distress consistent with a diagnosis of moderate depression and/or anxiety disorder*), 22-29.9 (*high level of distress*) and 30 or more (*possibility of very high or severe levels of distress*) (Sulaiman-Hill & Thompson, 2010). In the current study, we will use a score of >15.9 as an indication of moderate to high levels of psychological distress.

The K10 was found to be an accurate screener for common mental disorders in patients in India (AUC=.87-.89) (Patel et al., 2008), in Arabic-speaking Moroccans in The Netherlands (AUC=.88) (Fassaert et al., 2009) and in other studies in high income studies (Sulaiman-Hill & Thompson, 2010). Furthermore, the K10 strongly correlated with other validated measures for screening for common mental disorders in primary care patients in India (*r*=.68 with GHQ-12 and *r*=.84 with SRQ, respectively) (Patel et al., 2008).

**Primary outcome measure**

*HSCL-25: Psychological distress*

The Hopkins Symptoms Checklist (HSCL-25) consists of 25 items related to psychological distress (Mollica, Wyshak, De Marneffe, Khuon, & Lavelle, 1987; Parloff, Kelman, & Frank, 1954). Subscales can be calculated for depression 913 items) and anxiety symptoms (10 items). There are two items related to somatic symptoms. The items are rated on a 4-point Likert scale, with a well-validated cut-off score of 1.75 (Mollica et al., 1987; Nettelbladt, Hansson, Stefansson, Borgquist, & Nordstrom, 1993). However, this cut-off has not been found as accurate in non-Western populations (Ventevogel et al., 2007). For the current study, we will primarily look at the total score and its change from baseline to follow-up. Furthermore, to examine changes in caseness in depression, we will use a cut-off score for the depression subscale of 2.1, which has been found to be a valid cut-off point in a Lebanese populations (Mahfoud et al., 2013).

The Arabic version of HSCL-25 has been used in various studies (Al-Turkait, Ohaeri, El-Abbasi, & Naguy, 2011; Caspi, Saroff, Suleimani, & Klein, 2008; Kobeissi et al., 2012; Selmo, Koch, Brand, Wagner, & Knaevelsrud, 2016). In addition, the measure has been used in studies to the effectiveness of lay-counsellor delivered, transdiagnostic, psychological interventions (e.g., Murray et al., 2014) and in traumatized refugees in Norway, where the HSCL-25 correlated highly with other measures of mental health (Lavik, Hauff, Oivind, & Laake, 1999).

**Secondary outcome measures**

*PCL-5: PTSD symptoms*

Posttraumatic stress disorder (PTSD) symptoms during the past week according to de DSM-5 PTSD diagnosis will be measured using the PTSD Checklist for DSM-5 (PCL-5) (Weathers et al., 2013), which is a 20-item checklist corresponding with the 20 DSM-5 PTSD symptoms. Items are rated on a 0-4 scale and add up to a total severity score of 80, with higher scores indicating worse symptomatology. The measure has previously been used in adults in Gaza (Thabet, Tawahina, El Sarraj, & Vostanis, 2008)

*PSYCHLOPS: self-identified problems*

The Psychological Outcomes Profiles (PSYCHLOPS) scale is a patient-generated outcome measure as an indicator of change after therapy (Ashworth et al., 2004). PSYCHLOPS consists of four questions. It contains three domains: problems (2 questions), function (1 question), and wellbeing (1 question). Participants are asked to give free text responses to the problem and function domains. Responses are scored on an ordinal six-point scale producing a maximum score of 18 (six points per domain). PSYCHLOPS has been validated in primary care populations across several countries (Czachowski, Seed, Schofield, & Ashworth, 2011; Héðinsson, Kristjánsdóttir, Ólason, & Sigurðsson, 2013).

*CSRI schedule: cost of care*

The Client Service Receipt Inventory (CSRI) was developed for the collection of data on service utilization and related characteristics of people with mental disorders, as the basis for calculating the costs of care for mental health cost-effectiveness research. It has been used cross-culturally and is available for The Netherlands (Chisholm et al., 2000). It will be translated to Arabic by the VU Amsterdam research team.

*PG-13: prolonged grief*

The PG-13 is the most universally used index of prolonged grief. Prolonged grief symptoms were assessed using the PG-13, which is a 13-item self-report measure that indexes the core symptoms of prolonged grief disorder (PGD. Eleven items are rated on a 5-point scale and two items on a 2-item scale, providing a possible total score of 57 with higher score reflecting worse symptoms.

*PQ-*B: prodromal psychotic symptoms. This measured is a widely used index of early indications of psychosis. The PQ-B comprises 16 true or false items, and ask about levels of distress experienced for the endorsed items on a 4-point scale. Respondents who endorse ≥ 6 items are considered to be at risk for developing psychosis.

*APQ:* parenting behaviour

The APQ measures five major parenting constructs: (i) involvement (10 items), (ii) poor supervision and monitoring (10 items), (iii) positive parenting (6 items), (iv) inconsistent discipline (6 items), and (v) corporal punishment (3 items). Each item is scored on a 5-point scale, with higher scores indicating greater strength of the relevant subscale.

*PSC:* children’s mental health

The PSC comprises 35 items rated on a 3-point scale and yields a total score, as well as three subscale scores of attentional (5 items), internalizing (5 items), and externalizing (7 items) problems. Higher scores indicate more severe difficulties in the respective domain.

**Covariates**

*LEC: exposure to potentially traumatic events*

Previous stressor exposure will be assessed using the Life Events Checklist (Weathers et al., 2013). This is a widely used list of 17 experienced or witnessed events, such as rape, serious injury, combat exposure, or the sudden death of a loved one. Based upon qualitative assessment before the trial, the list of potentially traumatic events will be adapted to the Syrian refugee context, if necessary.

*PMLD: post-migration stressors*

Post-migration stressors will be assessed using a version of the Post-Migration Living Difficulties Checklist (PMLDC) (Silove, Sinnerbrink, Field, Manicavasagar, & Steel, 1997; Steel, Silove, Bird, McGorry, & Mohan, 1999) adapted to the Swiss context. This 17-item scale examines the extent to which post-migration challenges had been of concern to the individual over the past 12 months. Items are rated on a five-point scale, ranging from 0 (*not a problem*) to 4 (*a very serious problem*). Items scored at least 3 (*a serious problem*) are considered positive responses, yielding a total count of living difficulties. This scale has consistently been identified as a predictor of mental health among displaced populations (Nickerson, Bryant, Steel, Silove, & Brooks, 2010; Schweitzer et al., 2006; Steel et al., 2006) and has previously been used in Arabic speaking refugees (Nickerson et al., 2015; Schick et al., 2016). In the current research, we will assess post-migration challenges over the past month (at baseline) and the period since the last assessment (at post-intervention assessment and follow-up).

**Study Phase 3: Process evaluation of administering PM+ to adult Syrian refugees in Jordan**

We will explore the feasibility, challenges and successes in carrying out research activities as well as the PM+ intervention through comprehensive process monitoring (see Step 1 below) and semi-structured interviews with five peer-refugee counsellors (see Step 2 below). PM+ participants and their family members will be approached to evaluate the burden of completing the assessments and PM+, satisfaction with the intervention, and barriers and facilitators to adherence. In addition, five decision-makers with responsibilities for developing or implementing health policy, including the manager and two psychologists of Noor Al Hussein will be interviewed to obtain their perceptions of the benefits and challenges of integrating PM+ into Noor Al Hussein’s routine service provision.

*Step 1: Process monitoring and treatment fidelity*

Process monitoring includes review of counsellor records of sessions with clients; counsellor supervision records including intervention fidelity monitoring; and supervision of supervisors by intervention trainers. The data will be collected throughout the intervention delivery (see Table 1) and reviewed as it is collected, leading to an iterative process of intervention monitoring informing intervention delivery.

*Step 2: Semi-structured interviews for the evaluation of PM+*

Individual semi-structured interviews will be conducted with five participants from each category of: a) PM+ participants (study completers and drop-outs), b) family members of PM+ participants, c) counsellors, d) psychologists of Noor Al Hussein and e) local stakeholders with a role in policy development or implementation. The aim of these interviews is to explore the feasibility of scaling-up the implementation of PM+ within Jordan, and to explore intervention adherence and satisfaction in-depth. Interviews will follow a semi-structured interview guide (see ‘F1. Topic lists qualitative interviews Study Phase 3 and 5 version 1 dd 24-05-2017’) with key questions that are identified for exploration, with additional prompt questions to fully explore each question in depth.

Before taking part in any key informant interviews oral and written information about the study and its purpose will be provided by the interviewer to respondents. This will be done in Arabic for the PM+ participants and their family members, and counsellors; it will be done in Arabic for policy makers and psychologists of Noor Al Hussein. Immediately after informed consent, the interviews will start.

*Qualitative analysis*

All key informant data will be analyzed following thematic analysis. Findings from this phase of the study will be used to further refine intervention delivery and to inform the definitive (fully powered) RCT of Study Phase 4.

**Study Phase 4: Definitive randomized controlled trial (RCT) to evaluate the effectiveness and cost-effectiveness of the culturally adapted PM+ for Syrian refugees in Jordan**

Study Phase 4 will be carried out at the community level in Amman overseen by the Noor Al Hussein Foundation. Eligibility and inclusion criteria will be similar as to those in the exploratory RCT. Participants will include adult female Syrian refugees with a child between the ages of 10-16years who a) score above K-10 (score of >15.9), and b) score above 16 on the WHO Disability Assessment Schedule 2.0. These instruments are administered by a research assistant and are described under Study Phase 2 (‘Measurement instruments’).

Similar to Study Phase 2, individuals with imminent suicide risk will be excluded and referred for appropriate treatment and support. In addition, we will also exclude individuals with severe mental disorder (psychotic disorders, substance-dependence) or severe cognitive impairment (e.g., severe intellectual disability or dementia). We will screen for these exclusion criteria using the tools in the PM+ intervention manual (WHO, 2016, pp. 86-87). Individuals meeting the exclusion criteria will be referred to International Medical Corps or to local social service provision, depending upon their needs. If the patient agrees, the assessment results will be provided to the treating mental health professional with permission of the patient and the research team.

Oral and written informed consent will be obtained in the same way as in Study Phase 2. Following informed consent for screening (see informed consent procedure described under Study Phase 2) (see Screening Consent Form), the research assistant will record demographic characteristics and participants will be invited to complete the K10, the WHODAS 2.0, and the PM+ intervention manual suicide tool. No identifiable information will be recorded. If they score above both the cut-off scores of the K-10 (score of >15.9) and the WHODAS 2.0 (score of >16), they will be given oral and written information about participating in the RCT by the research assistant. At least 24 hours after, a research assistant will ask informed consent to participate in the trial (see Intervention Consent Form).

Next, the HSCL-25, the WHODAS 2.0, the Psychological Outcome Profiles instrument (PSYCHLOPS), the Life Events Checklist (LEC), the PTSD Checklist for DSM-5 (PCL-5), SDQ, and the Client Service Receipt Inventory (CSRI) will be administered.

If participants are not selected for the trial because they score below the cut-off scores for the K10 or the WHODAS 2.0, they will be provided feedback on their test outcomes and will be explained why they are not eligible for the study.

After the assessment, participants will be randomized by an independent research assistant, not involved in the assessments, in either the PM+ intervention (*n*=173) or the treatment as usual (TAU) control condition (*n*=173). Ass for the exploratory RCT, randomization will be performed using computerized software on a 1:1 basis by an independent researcher not involved in intervention delivery, clinical supervision, independent assessment or other aspects of the day-to-day running of the study. If randomized into the PM+ condition, participants will be allocated to an intervention group by the independent researcher who performed the randomization. Groups will comprise 6-8 women.

The post-intervention assessment (WHODAS 2.0, HSCL-25, PSYCHLOPS, LEC, PCL-5, PMLD, CSRI schedule, PG-13, PQ-B, APQ, PSC, and additional questions on perceived access to health services) will be scheduled seven weeks after the pre-intervention assessment (i.e., one week after the fifth PM+ session, see Figure 1). The two follow-up assessments will be conducted three and 12 months after the fifth PM+ session. All instruments of post-intervention assessment will be administered again at both follow-up assessments. The LEC will only be assessed at 12-months follow-up.

The HSCL-25 is the primary outcome measure; the WHODAS 2.0, PSYCHLOPS, PCL-5, PG-13, PQ-B, APQ, PSC, CSRI schedule, and perceived access to health services are the secondary outcome measures.

In case participants do not attend a scheduled assessment, they are called a maximum of five times (on different days) for scheduling a new appointment.

*Assessors*

All instruments will be administered by trained research staff blind to the allocation status of the participants (i.e., single-blinded trial). All assessors will receive a five-day training in administering the instruments, in general interview techniques, and in responding to participant distress (see Study Phase 2 for a more detailed description of the training).

*Measurement instruments*

See overview under Study Phase 2.

**Study Phase 5: Process evaluation of administering PM+ to adult Syrian refugees in Jordan**

The procedure described under Study Phase 3 will be repeated in Study Phase 5.

## Withdrawal of individual subjects

Subjects can leave the study at any time for any reason if they wish to do so without any consequences. The investigator can decide to withdraw a subject from the study for urgent medical reasons.

### Specific criteria for withdrawal (if applicable)

Not applicable for this study.

## Replacement of individual subjects after withdrawal

No new subjects will be included for each withdrawn subject.

## Follow-up of subjects withdrawn from treatment

If a subject decides to withdraw from the study, the investigator will ask for the reason. It will be enquired whether the subject wishes to withdraw from the study or from a specific time point only and so whether the subject can be re-contacted at a later time. Withdrawal from the study will have no effect on the regular treatment. Subjects who leave the study for medical reasons will be followed until the interfering condition has resolved or reached a stable state.

## Premature termination of the study

Not applicable.

# SAFETY REPORTING

## Temporary halt for reasons of subject safety

In accordance to section 10, subsection four, of the WMO, the sponsor will suspend the study if there is sufficient ground that continuation of the study will jeopardise subject health or safety. The sponsor will notify the accredited METC without undue delay of a temporary halt including the reason for such an action. The study will be suspended pending a further positive decision by the accredited METC. The investigator will take care that all subjects are kept informed.

## AEs and SAEs

### Adverse events (AEs)

Adverse events are defined as any undesirable experience occurring to a subject during the study, whether or not considered related to the trial procedure or the PM+ intervention. All adverse events reported spontaneously by the subject or observed by the investiga­tor or his staff will be recorded.

### Serious adverse events (SAEs)

A serious adverse event is any untoward medical occurrence or effect that

- results in death;
- is life threatening (at the time of the event);
- requires hospitalisation or prolongation of existing inpatients’ hospitalisation;
- results in persistent or significant disability or incapacity;
- is a congenital anomaly or birth defect; or
- any other important medical event that did not result in any of the outcomes listed above due to medical or surgical intervention but could have been based upon appropriate judgement by the investigator.

An elective hospital admission will not be considered as a serious adverse event.

The investigator will report all SAEs to the sponsor without undue delay after obtaining knowledge of the events, except for the following SAEs: Not applicable.

## Follow-up of adverse events

All AEs will be followed until they have abated, or until a stable situation has been reached. Depending on the event, follow-up may require additional tests or medical procedures as indicated, and/or referral to the general physician or a medical specialist.

SAEs need to be reported till end of study to UNSW, as defined in the protocol

## Safety Committee

The STRENGTHS’ Safety Board (SB) will monitor all ethical, legal and societal issues that arise within the STREGNTHS project .The SB will ensure that the trial and data collection are conducted in accordance with the International Conference on Harmonisation (ICH), the WHO Good Clinical Practice standards (GCP), Declaration of Helsinki (64th WMA General Assembly, Fortaleza, Brazil, October 2013), and (inter)national laws (e.g, Medical Research Involving Human Subjects Act (WMO)). In addition, the safety, rights and wellbeing of the participants and research staff members will be reviewed and interim analyses will be considered in case safety issues are (suspected to be) violated. The SB defined an incidental findings policy, which is added to this study protocol. Incidental findings within STRENGTHS refer to an extreme score on study instruments (questionnaires or interviews) that need additional follow-up. The policy describes who will identify such incidental findings, which additional professional expertise will be called in, on what moment this will be done, which findings will be reported to whom, and how it will be reported (face-to-face or by mail). In the case of political instability and armed conflict, the SB will assess the risks for research participants and staff, and take appropriate measures if needed. Other issues that will be considered include privacy and intellectual property rights. Relevant issues will be discussed periodically (on a six-month base) in a meeting, but if issues arise between these meetings, the SB will be requested to plan an additional meeting. The SB will submit a report with the periodic reports. The SB compromises three consortium members: dr. Egbert Sondorp, dr. Monique Pfaltz and dr. Marit Sijbrandij.

The management team and SB will ensure that all necessary actions will be undertaken to minimize risks and suggest necessary measures to counter these risks. Through efficient communication between the SB, overall management (Work Package 1), and leader of individual Work Packages, the consortium will ensure that mitigation measures will be undertaken in a timely and effective manner.

The advice(s) of the Safety Board will only be sent to the sponsor of the study. Should the sponsor decide not to fully implement the advice of the Safety Board, the sponsor will send the advice to the reviewing METC, including a note to substantiate why (part of) the advice of the SB will not be followed.

# STATISTICAL ANALYSIS

The statistical analyses for both the exploratory and the definitive RCT are described under section 8.1-8.2 below.

## Primary study parameter(s)

The statistical analyses for Study Phase 2 and 4 will be discussed together.

The statistical analysis of the exploratory RCT is planned to obtain estimates of drop-out rates to inform the definitive RCT (Objective 1). The statistical analysis of the definitive RCT is to estimate the effectiveness and cost-effectiveness of the PM+ intervention (Objective 3).

For both trials, the primary outcome will be summarized using number of subjects (*n*), minimum and maximum; and means, standard deviations (*SD*) for normally distributed data, or medians and inter-quartile ranges for non-normally distributed data. To measure comparisons at baseline between the two treatment groups *t*-tests (continuous variables) or chi-squared test (categorical variables) will be conducted for normally distributed data; Mann-Whitney tests will be conducted for continuous non-normally distributed data.

Both intention-to-treat (ITT) analysis, including all randomized participants (exploratory trial *n*=60; definitive trial *n*=410), and completers’ (PP) analysis will be carried out. The main conclusion in the Study Phase 2 report will be based on the ITT analysis of the primary outcome. A secondary analysis of the primary outcome will also be presented using the PP population. Similarly, to the exploratory trial, the main conclusion of the definitive trial will be based on the ITT analysis. The same analysis plan exists for the secondary outcomes measures. The APQ will be analyzed according to the respective subscale scores to reflect different features of positive and negative parenting. The PSC will be analyzed according to the subscale scores to determine the effects of intervention on different aspects of children’s mental health (i.e. attentional, internalizing, and externalizing problems). Exploratory analyses will also be conducted that potentially consider refugees’ mental health, parenting behavior, and changes in child mental health.

To estimate the treatment effect, a linear mixed model will be employed for the primary endpoint analysis, which will have treatment as fixed effects, baseline measurement of primary endpoint as covariate, and subject as random effects. The mean difference between two treatment arms at each visit/time together with its 95% confidence interval will be derived from the mixed model.

*Missing data*

Missing data will be treated as missing at random (MAR). No imputations of missing values will be made, as multilevel models can deal with missing data (Singer & Willett, 2003).

## Secondary study parameter(s)

*Economic outcome –* Health economic analysis will be conducted to determine the difference in costs and outcomes in the intervention arm as compared to the treatment as usual group. Primary analysis will be the total costs over the 12-month follow-up treatment period. Between-group comparison of mean costs will be completed using standard *t*-test with ordinary least squares regression used for adjusted analysis, with the validity of results confirmed using bootstrapping. De-identified data will be sent to the London School of Economics and Political Science, partner in STRENGTHS under Work Package 7, for the health economics analysis of the CSRI.

*Analysis of secondary outcomes with repeated measurements –* Additionally, a linear mixed model as mentioned for the primary outcome analysis will be carried out for analyzing the following clinical outcomes measured at baseline, post-intervention, three- and (for the definitive RCT only), and for the 12-month follow-up.

*Analysis of other secondary outcomes –* Continuous secondary outcomes will be analyzed in the same way as the primary endpoint analysis. For the analysis of binary outcomes, generalized mixed model will be employed with treatment as fixed effects, baseline measurement as covariate, and subject as random effect. The odds ratio between two treatment arms at each visit together with its 95% confidence interval will be derived from the generalized mixed model.

Changes in caseness of depression will be calculated for the PP sample using the recommended cut-off of >2.1 (Mahfoud et al., 2013) on the Depression subscale of the HSCL-25 and will be analyzed using a hierarchical logistic model with the same fixed and random effects as HLM models above, from which odds ratio of having a depression together with 95% CI at each time point will be derived.

*Corrections for multiple testing*

Models will be tested on α = .05; we will not apply a post-hoc correction to deal with problems associated with multiple testing, but instead report the number of tests that are carried out.

## Other study parameters

This study is preceded by qualitative interviews as part of Work Packages 2 and 3. The outcomes of these assessments will be used to make informed-decisions for potential mediators or moderators of PM+ treatment effectiveness.

*Treatment fidelity*: In order to determine whether the intervention-as-implemented does not differ from the intervention-as-designed, fidelity checklists filled out by VU Amsterdam research assistants are completed for a random sample, stratified on peer-refugee counsellor, of sessions/participants. Treatment fidelity will be analysed as manipulation check.

## Interim analysis (if applicable)

Interim analyses will be considered in case safety issues are (suspected to be) violated. See ‘Safety committee’.

# ETHICAL CONSIDERATIONS

## Regulation statement

This study will be conducted according to the principles of the Declaration of Helsinki (64th WMA General Assembly, Fortaleza, Brazil, October 2013), and in accordance with the International Conference on Harmonisation (ICH), the WHO Good Clinical Practice standards (GCP), and the NHMRC.

## Recruitment and consent

For details on the informed consent procedure, see ‘Study Procedures’.

Eligible participants will be adult (18 years or above) female Syrian refugees without acute medical conditions (see inclusion and exclusion criteria in paragraph 2.2 and 2.3). They will first be orally informed about the project by a research staff member and will be asked whether they agree that a member of the research team will provide them with further information about the research. Only if permission is given will a research assistant administer the screening. Participants will be free to decline to participate or withdraw at any time without affecting their routine care.

The informed consent process entails a two-step procedure;
*1. Informed consent for screening*Participants will first be orally informed about the project by research staff, and will be asked whether they agree that a member of the research team will provide them with further information about the study. Participants will be free to decline to participate or withdraw at any time. Respondents who decide to participate will be asked to complete a written consent form. For participants who are illiterate, witnessed oral consent and a thumb print in lieu of a signature will be sufficient. The witness will be a member of the research staff team.

*2. Informed consent for taking part in the PM+ trial*
If participants meet the eligibility criteria (K10 >15.9 and WHODAS 2.0 >16), they will be given oral and written information about participating in the RCT by the research assistant. At least 24 hours after, a research assistant will ask informed consent to participate in the trial (see consent form).Participants are allowed to withdraw from the study at any time after they have given their written consent.

## Objection by minors or incapacitated subjects (if applicable)

If a child decides to not participate, they are entitled to withdraw.

## Benefits and risks assessment, group relatedness

Participants randomized into the PM+ intervention group may benefit from their participation in terms of expected reductions in psychological distress. The risks associated with participation are estimated to be minimal, since PM+ reduced psychological distress in previous studies in Pakistan and Kenya (Bryant et al., 2016; A Rahman et al., n.d.; Atif Rahman, Hamdani, Awan, Bryant, Dawson, Khan, Mukhtar-ul-Haq Azeemi, et al., 2016). Participants in both the treatment and control group will not be withheld treatment as usual (note: the intervention group receives treatment as usual (TAU) with PM+, the control group will receive TAU only).

It is possible that participants experience (increased) stress during the PM+ sessions. The intervention will be supervised and strictly monitored (two-weekly) by experienced psychologists of Noor Al Hussein. If a participant deteriorates during the intervention period, (s)he can be referred within Noor Al Hussein and this will be monitored by the supervisors.

Participants may experience distress during the interviews. Administering the instruments is crucial to draw conclusions about the feasibility and credibility of the intervention. The assessors are trained by the research team. In case of an undesirable emotional reaction both during the intervention as well as during the follow-up assessments the researcher or a clinician will be available to provide support if necessary or desirable. When a participant has elevated symptoms at follow-up assessments, (s)he will be advised to contact his/her general practitioner (part of the TAU), who may refer the participant for continued or high-intensity treatment (i.e., stepped-care process leading to more specialized care high-intensity treatment).

## Incentives (if applicable)

Participants will receive $3JD per assessment.

# ADMINISTRATIVE ASPECTS, MONITORING AND PUBLICATION

## Handling and storage of data and documents

All data will be handled confidentially and will be coded by a code known only by the principle investigator (Prof Bryant) and main collaborator at Noor Al Hussein (Dr Atef Shwashreh).

*Qualitative data collection*

Before commencing a qualitative interview, the date of the interview and the interview number will be recorded. No identifying information will be collected during the qualitative interviews, with all data de-identified. Information will be transcribed in the Microsoft word program (secured with a password known to the research team only) and safely stored at the office of the principal investigator who coordinates the research at UNSW (Prof Bryant).

*Quantitative data collection*

Quantitative data will be coded and the identifying key (a list connecting names to numbers) will be kept in a separate, secure locked location in the coordinating researcher’s office (Prof Bryant). The data will be entered into a data-analytic computer program (e.g., SPSS), without the identifying key. Data will only be available to the members of the project group. The project group will analyze the data, and both positive and negative trial results will be disclosed. No attributable data will be used in publications. Results will be submitted for publication to peer-reviewed scientific journals.

The qualitative and quantitative data of all study phases, except for Study Phase 1, are stored for a period of 7 yearsThere are no conflicts of interest.

## Monitoring and Quality Assurance

Process monitoring is part of Study Phases 3 and 5, described in more detail under ‘Study Procedures’. It includes review of peer-refugee counsellor records of PM+ sessions, supervision records for including intervention fidelity monitoring and supervision and supervision of supervisors by intervention trainers. The supervision of the peer-refugee counsellors will be scheduled every two weeks. The supervision of supervisors by the PM+ trainers will be scheduled monthly (1-2 hours). This is similar to the procedure used by Rahman and colleagues (2016) for the PM+ trial in Pakistan.

Treatment fidelity is one of the outcome measures. The data will be collected throughout the intervention delivery (see Table 1).

Monitoring of the assessments will be the responsibility of the main investigator (Prof Bryant). If indicated, psychologists will conduct full assessments where there are questions about the capacity of an individual to carry out their role effectively. This oversight will help ensure that any potential concerns about the capacity of assessors to carry out their roles is picked up and responded to.

## Amendments

Amendments are changes made to the research after a favourable opinion by the accredited METC has been given. All amendments will be notified to the METC that gave a favourable opinion.

## Annual progress report

The sponsor/investigator will submit a summary of the progress of the trial to the accredited METC once a year. Information will be provided on the date of inclusion of the first subject, numbers of subjects included and numbers of subjects that have completed the trial, serious adverse events/ serious adverse reactions, other problems, and amendments.

## Temporary halt and (prematurely) end of study report

The investigator/sponsor will notify the accredited METC of the end of the study within a period of eight weeks. The end of the study is defined as the last patient’s last visit.

The sponsor will notify the METC immediately of a temporary halt of the study, including the reason of such an action.

In case the study is ended prematurely, the sponsor will notify the accredited METC within 15 days, including the reasons for the premature termination.

Within one year after the end of the study, the investigator/sponsor will submit a final study report with the results of the study, including any publications/abstracts of the study, to the accredited METC.

## Public disclosure and publication policy

The trial will be registered in a public trial registry (the Australian and New Zealand Clinical Trials Registry) before the first patient is recruited. The results of this study will be submitted for publication in international, peer-reviewed journals.

#

# REFERENCES

Al-Turkait, F. A., Ohaeri, J. U., El-Abbasi, A. H. M., & Naguy, A. (2011). Relationship between symptoms of anxiety and depression in a sample of arab college students using the Hopkins Symptom Checklist 25. *Psychopathology*, *44*(4), 230–241. https://doi.org/10.1159/000322797

Ashworth, M., Shepher, M., Christey, J., Matthews, V., Wright, K., Parmentier, H., … Godfrey, E. (2004). A client-generated psychometric instrument: the development of “PSYCHLOPS.” *Counseling and Psychotherapy Research*, *4*(2), 27–32. https://doi.org/10.1093/astrogeo/atw101

Barnes, C. S., Brown, R. P., & Tamborski, M. (2012). Living dangerously: culture of honor, risk-taking, and the nonrandomness of “accidental” deaths. *Social Psychological and Personality Science*, *3*, 100–107.

Bolton, P., Tol, W. A., & Bass, J. (2009). Combining qualitative and quantitative research methods to support psychosocial and mental health programmes in complex emergencies. *Intervention*, *7*(3), 181–186. https://doi.org/10.1097/WTF.0b013e32831e12d4

Bryant, R., Dawson, K., Schafer, A., Sijbrandij, M., & van Ommeren, M. (2016). Randomized controlled trial of a brief mental health intervention for women affected by gender-based violence. *Manuscript under Review*.

Cartwright, K., El-Khani, A., Subryan, A., & Calam, R. (2015). Establishing the feasibility of assessing the mental health of chidlren displaced by the Syrian conflict. *Global Mental Health*, *2*. https://doi.org/10.1017/gmh.2015.3

Caspi, Y., Saroff, O., Suleimani, N., & Klein, E. (2008). Trauma exposure and posttraumatic reactions in a community sample of bedouin members of the Israel defense forces. *Depression and Anxiety*, *25*(8), 700–707. https://doi.org/10.1002/da.20449

Chisholm, D., Knapp, M., Knudsen, H., Amaddeo, F., Gaite, L., & van Wijngaarden, B. (2000). Client Socio-Demographic and Service Receipt Inventory-European Version: development of an instrument for international research. EPSILON Study 5. European Psychiatric Services: Inputs Linked to Outcome Domains and Needs. *British Journal of Psychiatry*, *39*, s28-33.

Craig, P., Dieppe, P., Macintyre, S., Michie, S., Nazareth, I., & Petticrew, M. (2013). Developing and evaluating complex interventions: The new medical research council guidance. *International Journal of Nursing Studies*, *50*(5), 587–592. https://doi.org/10.1016/j.ijnurstu.2012.09.010

Czachowski, S., Seed, P., Schofield, P., & Ashworth, M. (2011). Measuring psychological change during cognitive behaviour therapy in primary care: a polish study using “PSYCHLOPS” (Psychological Outcome Profiles). *PLoS ONE*, *6*(12), e27378–e27378. https://doi.org/10.1371/journal.pone.0027378

Dawson, K. S., Bryant, R. A., Harper, M., Kuowei Tay, A., Rahman, A., Schafer, A., & Van Ommeren, M. (2015). Problem Management Plus (PM+): A WHO transdiagnostic psychological intervention for common mental health problems. *World Psychiatry*, *14*(3), 354–357. https://doi.org/10.1002/wps.20255

Dua, T., Barbui, C., Clark, N., Fleischmann, A., Poznyak, V., van Ommeren, M., … Saxena, S. (2011). Evidence-based guidelines for mental, neurological, and substance use disorders in low- and middle-income countries: Summary of WHO recommendations. *PLoS Medicine*, *8*(11), e1001122. https://doi.org/10.1371/journal.pmed.1001122

Fassaert, T., de Wit, M. A. S., Tuinebreijer, W. C., Wouters, H., Verhoeff, A. P., Beekman, A. T. F., & Dekker, J. (2009). Psychometric properties of an interviewer-administered version of the Kessler Psychological Distress scale (K10) among Dutch, Moroccan and Turkish respondents. *International Journal of Methods in Psychiatric Research*, *18*(3), 159–168.

Goldberg, D., & Williams, P. (1988). *User’s guide to the General Health Questionnaire*. Windsor: NFER-Nelson.

Gornall, J. (2015). Healthcare for Syrian refugees. *BMJ*, *315*(August), h4150. https://doi.org/10.1136/bmj.h4150

Hassan, G., Kirmayer, L., Mekki-Berrada, A., Quosh, C., el Chammay, R., Deville-Stoetzel, J. B., … Ventevogel, P. (2015). *Culture, Context and the Mental Health and Psychsocial Wellbeing of Syrians: A Review for Mental Health and Psychosocial Support staff working with Syrian effected by Armed Conflict*. Geneva.

Héðinsson, H., Kristjánsdóttir, H., Ólason, D. Þ., & Sigurðsson, J. F. (2013). A validation and replication study of the patient-generated measure PSYCHLOPS on an Icelandic clinical population. *European Journal of Psychological Assessment*, *29*(2), 89–95. https://doi.org/10.1027/1015-5759/a000136

Kessler, R. C., Andrews, G., Colpe, L. J., Hiripi, E., Mroczek, D. K., Normand, S.-L. T., … Zaslavsky, A. M. (2002). Short screening scales to monitor population prevalences and trends in non-specific psychological distress. *Psychological Medicine*, *32*, 959–976. https://doi.org/10.1017/ S0033291702006074

Kobeissi, L., Mahfoud, Z., Khoury, B., El Kak, F., Ghantous, Z., Khawaja, M., … Peters, T. J. (2012). The Relaxation Exercise and Social Support Trial (RESST): a community-based randomized controlled trial to alleviate medically unexplained vaginal discharge symptoms. *BMC Psychiatry*, *12*(1), 195. https://doi.org/10.1186/1471-244X-12-195

Lavik, N. J., Hauff, E., Oivind, S., & Laake, P. (1999). The use of self-reports in psychiatric studies of traumatized refugees: Validation and analysis of HSCL-25. *Nordic Journal of Psychiatry*, *53*(1), 17–20. https://doi.org/10.1080/080394899426666

Mahfoud, Z., Kobeissi, L., Peters, T. J., Araya, R., Ghantous, Z., & Khoury, B. (2013). The Arabic validation of the Hopkins Symptoms Checklist-25 against MINI in a disadvantaged suburb of Beirut, Lebanon. *International Journal of Educational and Psychological Assessment*, *13*(1), 17–33.

Ministry of Security and Justice. (2016). *Asylum Trends. Monthly Report on Asylum Applications in The Netherlands*. Rijswijk. Retrieved from https://ind.nl/Documents/ASylum Trends December 2016.pdf

Mollica, R. F., Wyshak, G., De Marneffe, D., Khuon, F., & Lavelle, J. (1987). Indochinese versions of the Hopkins Symptom Checklist-25: A screening instrument for the psychiatric care of refugees. *American Journal of Psychiatry*, *144*(4), 497–500.

Morgan, J. (2015). Agencies struggle with Europe’s complex refugee crisis. *The Lancet*, *386*, 2042–2043. https://doi.org/10.1016/S0140-6736(15)01032-6

Murray, L. K., Dorsey, S., Bolton, P., Jordans, M. J., Rahman, A., Bass, J., & Verdeli, H. (2011). Building capacity in mental health interventions in low resource countries: an apprenticeship model for training local providers. *International Journal of Mental Health Systems*, *5*(1), 30. https://doi.org/10.1186/1752-4458-5-30

Murray, L. K., Dorsey, S., Haroz, E., Lee, C., Alsiary, M. M., Haydary, A., … Bolton, P. (2014). A common elements treatment approach for adult mental health problems in low- and middle-income countries. *Cognitive and Behavioral Practice*, *21*(2), 111–123. https://doi.org/10.1016/j.cbpra.2013.06.005

Nettelbladt, P., Hansson, L., Stefansson, C. G., Borgquist, L., & Nordstrom, G. (1993). Test characteristics of the Hopkins Symptom Check List-25 (HSCL-25) in Sweden, using the present state examination (PSE-9) as a caseness criterion. *Social Psychiatry Psychiatric Epidemiology*, *28*(3), 130–133.

Parloff, M. B., Kelman, H. C., & Frank, J. D. (1954). Comfort, effectiveness, and self-awareness as criteria of improvement in psychotherapy. *The American Journal of Psychiatry*, *111*(5), 343–352. https://doi.org/10.1176/ajp.111.5.343

Patel, V., Araya, R., Chowdhary, N., King, M., Kirkwood, B., Nayak, S., … Weiss, H. A. (2008). Detecting common mental disorders in primary care in India: a comparison of five screening questionnaires. *Psychological Medicine*, *38*(2), 221–228. https://doi.org/10.1017/S0033291707002334

Rahman, A., Hamdani, S. U., Awan, N. R., Bryant, R. A., Dawson, K. S., Khan, M. F., … Van Ommeren, M. (2016). Effect of a multicomponent behavioral intervention in adults impaired by psychological distress in a conflict-affected area of Pakistan. A randomized clinical trial. *JAMA*, *316*(24), 2609–2617. https://doi.org/10.1001/jama.2016.17165

Rahman, A., Hamdani, S. U., Awan, N. R., Bryant, R. A., Dawson, K. S., Khan, M. F., … Van Ommeren, M. (2016). Effect of a multicomponent behavioral intervention in adults ipairment by psychological distress in a conflict-affected area of Pakistan. A randomized controlled trial. *JAMA*, *316*(24), 2609–2617. https://doi.org/10.1001/jama.2016.17165

Rahman, A., Riaz, N., & Dawson, K. (n.d.). Problem Management Plus (PM+): pilot trial of a WHO transdiagnostic psychological intervention in conflict-affected Pakistan. World psychiatry : official journal of the World Psychiatric Association. in press. *World Psychiatry: Official Journal of the World Psychiatric Association*.

Selmo, P., Koch, T., Brand, J., Wagner, B., & Knaevelsrud, C. (2016). Psychometric Properties of the Online Arabic Versions of BDI-II, HSCL-25, and PDS. *European Journal of Psychological Assessment*, *Advance on*, 1–9. https://doi.org/10.1027/1015-5759/a000367

Singer, J. D., & Willett, J. B. (2003). *Applied Longitudinal Data Analysis: Modeling Change and Event Occurrence*. New York: Oxford University Press, Inc.

Slobodin, O., & Jong, J. T. V. M. De. (2015). Mental health interventions for traumatized asylum seekers and refugees: what do we know about their efficacy? *International Journal of Social Psychiatry*, *61*, 17–26. https://doi.org/10.1177/0020764014535752

Steel, Z., Chey, T., Silove, D., Marnane, C., Bryant, R., & van Ommeren, M. (2009). *Policy brief on migration and health: mental health care for refugees*. Copenhagen.

Stenmark, H., Catani, C., Neuner, F., Elbert, T., & Holen, A. (2013). Treating PTSD in refugees and asylum seekers within the general health care system. A randomized controlled multicenter study. *Behaviour Research and Therapy*, *51*(10), 641–647. https://doi.org/10.1016/j.brat.2013.07.002

Sulaiman-Hill, C. M., & Thompson, S. C. (2010). Selecting instruments for assessing psychological wellbeing in Afghan and Kurdish refugee groups. *BMC Research Notes*, *3*, 237. https://doi.org/10.1186/1756-0500-3-237

Thabet, A. A. M., Abed, Y., & Vostanis, P. (2004). Comorbidity of PTSD and depression among refugee children during war conflict. *Journal of Child Psychology and Psychiatry and Allied Disciplines*, *45*(3), 533–542. https://doi.org/10.1111/j.1469-7610.2004.00243.x

Thabet, A. A., Tawahina, A. A., El Sarraj, E., & Vostanis, P. (2008). Exposure to war trauma and PTSD among parents and children in the Gaza strip. *European Child and Adolescent Psychiatry*, *17*(4), 191–199. https://doi.org/10.1007/s00787-007-0653-9

Tol, W. a, Barbui, C., & van Ommeren, M. (2013). Management of acute stress, PTSD, and bereavement: WHO recommendations. *JAMA*, *310*(5), 477–478. https://doi.org/10.1001/jama.2013.166723

UNHCR. (2016). UNHCR. Retrieved March 29, 2016, from http://data.unhcr.org/syrianrefugees/syria.php

UNICEF. (2016). Syrian refugees and other affected populations in Egypt, Iraq, Jordan, Lebanon and Turkey.

Ventevogel, P., De Vries, G., Scholte, W. F., Shinwari, N. R., Faiz, H., Nassery, R., … Olff, M. (2007). Properties of the Hopkins symptom checklist-25 (HSCL-25) and the Self-Reporting Questionnaire (SRQ-20) as screening instruments used in primary care in Afghanistan. *Social Psychiatry and Psychiatric Epidemiology*, *42*(4), 328–335. https://doi.org/10.1007/s00127-007-0161-8

Weathers, F., Blake, D., Schnurr, P., Kaloupek, D., Marx, B., & Keane, T. (2013). The Life Events Checklist for DSM-5 (LEC-5). Retrieved March 3, 2017, from http://www.ptsd.va.gov/professional/assessment/te-measures/life_events_checklist.asp

Weathers, F. W., Litz, B. T., Keane, T. M., Palmieri, P. A., Marx, B. P., & Schnurr, P. P. (2013). The PTSD Checklist for DSM-5 (PCL-5).

WHO. (2010a). *Measuring Health and Disability: Manual for WHO Disability Assessment Schedule WHODAS 2.0*. (T. B. Üstun, N. Kostansjek, S. Chatterji, & J. Rehm, Eds.), *World Health Organization*. Geneva: WHO. Retrieved from https://books.google.com/books?hl=en&lr=&id=h9fhLNiaRTgC&pgis=1

WHO. (2010b). *mhGAP intervention guide for mental, neurological and substance use disorders in non-specialized health settings: mental health gap action programme (mhGAP).* Geneva: WHO. Retrieved from http://scholar.google.com/scholar?hl=en&btnG=Search&q=intitle:mhGAP+Intervention+Guide#1%5Cnhttp://scholar.google.com/scholar?hl=en&btnG=Search&q=intitle:mhGAP+intervention+guide#1%5Cnhttp://www.ncbi.nlm.nih.gov/pubmed/23741783

WHO. (2010c). World Health Organization Disability Assessment Schedule II (WHODAS II). Geneva, Switzerland: World Health Organization.

WHO. (2013). *Guidelines for the management of conditions specifically related to stress*. Geneva.

WHO. (2016). Problem Management Plus (PM+): Individual psychological help for adults impaired by distress in communities exposed to adversity. Geneva: WHO.

1. Synthesis and dissemination of PM+ for Syrian refugees in European countries and countries bordering Syria is part of Work Package 7 of STRENGTHS and will be completed by the London School of Economics and Political Science. [↑](#footnote-ref-1)
2. Psychologists from i-psy will receive the training-of-trainers (TOT) program. The TOT is part of Work Package 3 of STRENGTHS and will be completed by the Danish Red Cross. [↑](#footnote-ref-2)
