# Supplementary material for: Effectiveness of a brief group behavioral intervention for common mental disorders in Syrian refugees in Jordan: A randomized controlled trial
Source: PLoS Med. 2022 Mar 17;19(3):e1003949. doi: 10.1371/journal.pmed.1003949 (PMC8929659; doi:10.1371/journal.pmed.1003949)
Supplement: S1 Table — (DOCX) [file pmed.1003949.s003.docx]

Table S1. Participant characteristics of participants who were retained and lost at follow-up

|  | Retained at 3-month follow-up  (n = 357) | Not Retained  (n = 53) | t/χ^2^ | *P* |
| --- | --- | --- | --- | --- |
| Female, n (%) | 265 (74.2) | 35 (66.0) | 1.58 | 0.21 |
| Age, years (SD) | 40.04 (7.04) | 40.00 (6.38) | -0.38 | 0.97 |
| Married, n (%) |  |  | 4.21 | 0.24 |
| Single | 0 (0) | 0 (0) |  |  |
| Married | 327 (91.6) | 49 (92.5) |  |  |
| Separated | 15 (4.2) | 1 (1.9) |  |  |
| Divorced | 3 (0.8) | 2 (3.8) |  |  |
| Widowed | 12 (3.4) | 1 (1.9) |  |  |
| Education |  |  | 9.46 | 0.05 |
| None | 83 (23.2) | 19 (35.8) |  |  |
| Basic certificate | 21 (39.6) | 212 (59.4) |  |  |
| Technical certificate | 8 (15.1) | 29 (8.1) |  |  |
| Secondary education | 3 (55.7) | 26 (7.3) |  |  |
| University | 2 (3.8) | 7 (2.0) |  |  |
| Time Since Leaving Syria |  |  | 0.96 | 0.62 |
| Less than 4 years | 84 (23.5) | 11 (20.8) |  |  |
| 5 – 6 years | 143 (40.1) | 19 (35.8) |  |  |
| 7 – 9 years | 130 (36.4) | 23 (43.4) |  |  |
| HSCL-25 Depression | 35.67 (9.15) | 37.07 (8.49) | 1.05 | 0.29 |
| HSCL-25 Anxiety | 24.90 (6.27) | 24.98 (5.67) | 0.09 | 0.93 |
| PCL | 26.07 (14.50) | 28.22 (15.00) | 1.00 | 0.32 |
| WHODAS | 23.67 (4.90) | 24.11 (5.64) | 0.60 | 0.55 |
| PSYCHLOPS | 16.14 (3.77) | 15.81 (4.36) | -0.58 | 0.56 |
| PG-13 | 28.61 (9.87) | 28.37 (11.05) | -0.12 | 0.90 |
| PQ | 13.38 (2.94) | 13.11 (3.13) | -0.61 | 0.54 |
| Alabama Involvement | 34.39 (8.84) | 37.27 (7.46) | 2.23 | 0.03 |
| Alabama Positive Parenting | 24.12 (5.01 ) | 25.91 (4.02) | 2.45 | 0.02 |
| Alabama Supervision | 14.78 (4.58) | 15.58 (6.09) | 1.13 | 0.26 |
| Alabama Discipline | 15.18 (4.06) | 14.71 (4.16) | -0.77 | 0.26 |
| Alabama Punish | 6.22 (2.69) | 6.19 (2.72) | -0.07 | 0.95 |
| PSC Attentional Problems | 9.26 (2.19) | 8.58 (2.22) | -1.99 | 0.05 |
| PSC Internalising | 8.30 (1.58) | 8.19 (1.48) | -0.47 | 0.64 |
| PSC Externalising | 10.64 (1.64) | 10.56 (1.29) | -0.33 | 0.74 |

Abbreviations: gPM+ = Group Problem Management Plus. EUC = Enhanced usual care. Abbreviations. EUC = Enhanced usual care; LS = Least Square; HSCL = Hopkins Symptom Checklist (depression subscale score range: 10-40; anxiety subscale score range: 15-60;

higher scores indicate elevated anxiety or depression); WHODAS = WHO Disability Assessment Schedule (total score range: 0-48; higher scores indicate more severe impairment); PCL-5 = Posttraumatic Stress Disorder Checklist (total score range: 0-80; higher scores indicate more severe PTSD severity); PSYCHLOPS = Psychological Outcomes Profiles (total score range: 0-20; higher scores indicate poorer outcome); PG-13 = Prolonged Grief Disorder 13 (total score range: 11-57; higher scores indicate poorer outcome). PQ = Prodromal Questionnaire (total score range: 0-64; higher scores indicate poorer outcome); Alabama Parenting Questionnaire (Parental Involvement subscale score range: 10-50; Positive Parent subscale score range: 6-30; Supervision subscale score range 10-50; Discipline subscale score range 6-30; Punishment subscale score range 3-15; higher scores indicate elevated parental involvement, positive parenting, supervision, discipline, and punishment). Pediatric Symptom Checklist is child’s self-report (PSC; Attention Problems subscale score range: 0-10; Internalising subscale score range: 0-10; Externalising subscale score range: 0-14). Effect size was calculated by the difference in least square means between intervention and EUC from mixed model divided by the pooled standard deviation.
