# Supplementary material for: Effectiveness of a brief group behavioral intervention for common mental disorders in Syrian refugees in Jordan: A randomized controlled trial
Source: PLoS Med. 2022 Mar 17;19(3):e1003949. doi: 10.1371/journal.pmed.1003949 (PMC8929659; doi:10.1371/journal.pmed.1003949)
Supplement: S2 Table — (DOCX) [file pmed.1003949.s004.docx]

S2 Table. Minimally Important Differences (MID) for Primary and Secondary Outcomes

|  | Χ^2^ (*p* value) | gPM+, n (%)  (n = 204) | EUC, n (%)  (n = 206) | Total, n (%) |
| --- | --- | --- | --- | --- |
|  |  |  |  |  |
| HSCL - Depression | 6.67 (.036) |  | | |
| Positive MID change |  | 102 (50.0) | 89 (43.2) | 191 (46.6) |
| No MID change |  | 49 (24.0) | 73 (35.4) | 122 (29.8) |
| Worsened MID change |  | 17 (8.4) | 27 (13.2) | 44 (10.7) |
| HSCL - Anxiety | 1.88 (.39) |  | | |
| Positive MID change |  | 110 (53.9) | 130 (63.1) | 240 (58.5) |
| No MID change |  | 28 (13.7) | 35 (17.0) | 63 (15.4) |
| Worsened MID change |  | 17 (8.3) | 27 (13.1) | 54 (13.2) |
| WHODAS | 6.40 (.04) |  | | |
| Positive MID change |  | 128 (76.2) | 125 (66.1) | 253 (70.9) |
| No MID change |  | 26 (15.5) | 50 (26.5) | 76 (21.3) |
| Worsened MID change |  | 14 (8.3) | 14 (7.4) | 28 (7.8) |
| PCL | 0.25 (.88) |  | | |
| Positive MID change |  | 117 (69.6) | 127 (67.2) | 244 (68.3) |
| No MID change |  | 39 (23.2) | 47 (24.9) | 86 (24.1) |
| Worsened MID change |  | 12 (7.1) | 15 (7.9) | 27 (7.6) |
| PG-13 | 0.16 (.92) |  | | |
| Positive MID change |  | 57 (57.6) | 58 (56.9) | 115 (57.2) |
| No MID change |  | 33 (33.3) | 33 (32.4) | 66 (32.8) |
| Worsened MID change |  | 9 (9.1) | 11 (10.8) | 20 (10.0) |
| PQ | 5.29 (.07) |  | | |
| Positive MID change | 100 (59.5) | 100 (52.9) | 200 (56.0) | 100 (59.5) |
| No MID change | 55 (32.7) | 58 (30.7) | 113 (31.7) | 55 (32.7) |
| Worsened MID change | 13 (7.7) | 31 (16.4) | 44 (12.3) | 13 (7.7) |
| PSYCHLOPS | 6.23 (.05) |  | | |
| Positive MID change |  | 14 (8.3) | 17 (9.0) | 31 (8.7) |
| No MID change |  | 70 (41.7) | 100 (52.9) | 170 (47.6) |
| Worsened MID change |  | 84 (50.0) | 72 (38.1) | 156 (43.7) |
| Alabama Parenting: Involvement | 0.21 (.90) |  | | |
| Positive MID change |  | 76 (45.5) | 92 (50.3) | 168 (48.0) |
| No MID change |  | 57 (34.1) | 58 (31.7) | 115 (32.9) |
| Worsened MID change |  | 34 (20.4) | 33 (18.0) | 67 (19.1) |
| Alabama Parenting: Positive Parenting | 0.82 (.66) |  | | |
| Positive MID change |  | 65 (40.9) | 77 (43.3) | 142 (42.1) |
| No MID change |  | 51 (32.1) | 54 (30.3)) | 105 (31.2) |
| Worsened MID change |  | 43 (27.0) | 47 (26.4) | 90 (26.7) |
| Alabama Parenting: Supervision | 2.59 (.27) |  | | |
| Positive MID change |  | 72 (43.4) | 81 (43.5) | 153 (43.5) |
| No MID change |  | 68 (41.0) | 86 (46.2) | 154 (43.8) |
| Worsened MID change |  | 26 (15.7) | 19 (10.2) | 45 (12.8) |
| Alabama Parenting: Discipline | 4.66 (.10) |  |  |  |
| Positive MID change |  | 96 (57.8) | 92 (50.3) | 188 (53.5) |
| No MID change |  | 36 (21.7) | 35 (19.1) | 71 (20.3) |
| Worsened MID change |  | 34 (20.5) | 56 (30.6) | 90 (25.8) |
| Alabama Parenting: Punishment | 3.60 (.16) |  | | |
| Positive MID change |  | 47 (35.8) | 66 (50.8) | 113 (44.8) |
| No MID change |  | 55 (45.1) | 40 (30.8) | 95 (37.7) |
| Worsened MID change |  | 20 (16.4) | 24 (18.5) | 44 (17.5) |
| PSC: Internalising Problems | 1.12 (.57) |  | | |
| Positive MID change |  | 53 (36.8) | 73 (44.0) | 126 (40.6) |
| No MID change |  | 66 (45.8) | 65 (39.2) | 131 (42.3) |
| Worsened MID change |  | 25 (17.4) | 28 (16.9) | 53 (17.1) |
| PSC: Externalising Problems | 0.65 (.72) |  | | |
| Positive MID change |  | 65 (45.5) | 81 (50.6) | 146 (48.2) |
| No MID change |  | 35 (24.5) | 32 (20.0) | 67 ()22.1 |
| Worsened MID change |  | 43 (30.1) | 47 (29.4) | 90 (29.7) |
| PSC: Attentional Problems | 1.80 (.41) |  | | |
| Positive MID change |  | 65 (45.5) | 74 (45.1) | 139 (45.3) |
| No MID change |  | 36 (25.2) | 36 (22.0) | 72 (23.5) |
| Worsened MID change |  | 42 (29.4) | 54 (32.9) | 96 (31.3) |

*Abbreviations.* gPM+ = Group Problem Management Plus; EUC = Enhanced Usual Care; HSCL = Hopkins Symptom Checklist; WHODAS = WHO Disability Assessment Schedule; PCL-5 = Posttraumatic Stress Disorder Checklist; PSYCHLOPS = Psychological Outcomes Profiles; PG-13 = Prolonged Grief Disorder 13; PQ = Prodromal Questionnaire; Alabama Parenting = Alabama Parenting Questionnaire; PSC = Pediatric Symptom Checklist.
