# Supplementary material for: Effectiveness of a brief group behavioral intervention for common mental disorders in Syrian refugees in Jordan: A randomized controlled trial
Source: PLoS Med. 2022 Mar 17;19(3):e1003949. doi: 10.1371/journal.pmed.1003949 (PMC8929659; doi:10.1371/journal.pmed.1003949)
Supplement: S3 Table — (DOCX) [file pmed.1003949.s005.docx]

S3 Table. Summary statistics and results from mixed model analysis of primary and secondary outcomes for participants

who completed three-month assessment

|  | | Descriptive statistics | | Mixed model analysis | | | |
| --- | --- | --- | --- | --- | --- | --- | --- |
| Primary and secondary outcomes | Visit | gPM+ (n = 168) | EUC (n = 189) | | Difference in LS mean (95%CI) | P-value | Effect size^a^ |
|  |  | Estimated Mean (SE) | Estimated Mean (SE) | |  |  |  |
| HSCL-25 Depression | Baseline | 36.43 (.70) | 35.00 (.67) | |  |  |  |
|  | 6-week | 29.80 (.76) | 32.30 (.72) | | 3.93 (1.95, 5.91) | .001 | 0.44 |
|  | 3 months | 28.94 (.74) | 31.20 (.70) | | 3.70(1.88, 5.52) | .001 | 0.41 |
| HSCL-25 Anxiety | Baseline | 24.86 (.48) | 24.93 (.46) | |  |  |  |
|  | 6-week | 20.49 (.52) | 21.92 (.50) | | 1.37 (-0.11, 2.86) | .07 | 0.23 |
|  | 3 months | 20.05 (.51) | 19.63 (.48) | | -.49 (-2.05, 1.08) | .54 | -.17 |
| WHODAS | Baseline | 23.51 (.38) | 23.82 (.36) | |  |  |  |
|  | 6-week | 15.12 (.62) | 15.49 (.61) | | 0.06 (-1.70, 1.83) | .94 | 0.01 |
|  | 3 months | 16.36 (.58) | 14.78 (.56) | | -1.89 (-3.58, -0.20) | .03 | -0.37 |
| PCL-5 | Baseline | 25.79 (1.12) | 26.32 (1.06) | |  |  |  |
|  | 6-week | 16.09 (1.10) | 17.62 (1.05) | | 0.99 (-2.45, 4.43) | 0.56 | 0.07 |
|  | 3 months | 10.25 (1.03) | 10.25 (.97) | | -0.53 (-3.95, 2.89) | 0.76 | -0.04 |
| PSYCHLOPS | Baseline | 16.48 (.27) | 15.72 (.27) | |  |  |  |
|  | 6-week | 13.34 (.36) | 13.67 (.35) | | 1.09 (0.19, 1.98) | 0.02 | 0.29 |
|  | 3 months | 13.46 (.34) | 13.59 (.33) | | 0.89 (0.07, 1.69) | .03 | 0.23 |
| ‏PG-13 | Baseline | 28.14 (.99) | 29.03 (.97) | |  |  |  |
|  | 6-week | 27.07 (1.09) | 27.51 (1.09) | | -0.24 (-3.75, 2.86) | 0.79 | -0.02 |
|  | 3 months | 20.60 (.77) | 21.31 (.76) | | -0.18 (-3.11, 2.75) | 0.90 | -0.02 |
| PQ | Baseline | 13.35 (.23) | 13.40 (.21) | |  |  |  |
|  | 6-week | 14.87 (.16) | 14.40 (.15) | | -0.52 (-1.20, 0.17) | 0.14 | -0.19 |
|  | 3 months | 15.08 (.14) | 14.95 (.13) | | -0.19 (-0.83, 0.47) | 0.58 | -0.07 |
| Alabama Involvement | Baseline | 34.25 (.69) | 34.51 (.65) | |  |  |  |
|  | 6-week | 33.66 (.69) | 32.87 (.66) | | -1.05 (-3.23, 1.13) | 0.35 | -0.12 |
|  | 3 months | 31.89 (.65) | 31.79 (.61) | | -0.36 (-2.64, 1.92) | 0.76 | -0.04 |
| Alabama Supervision | Baseline | 14.73 (.36) | 14.84 (.34) | |  |  |  |
|  | 6-week | 12.86 (.32) | 13.51 (.30) | | -0.09 (-1.15, 0.98) | 0.88 | -0.02 |
|  | 3 months | 12.39 (.25) | 12.42 (.24) | | 0.15 (-0.91, 1.99) | 0.79 | 0.03 |
| Alabama Positive Parenting | Baseline | 23.80 (.39) | 24.43 (.37) | |  |  |  |
|  | 6-week | 23.40 (.37) | 23.50 (.35) | | -0.52 (-1.73, 0.68) | 0.39 | -.11 |
|  | 3 months | 21.82 (.35) | 22.24 (.33) | | -.21 (-1.40, 0.97) | 0.72 | -.04 |
| Alabama Discipline | Baseline | 15.60 (.31) | 14.80 (.30) | |  |  |  |
|  | 6-week | 13.46 (.28) | 13.56 (.27) | | 0.90 (-0.15, 1.96) | 0.09 | 0.22 |
|  | 3 months | 13.01 (.28) | 13.59 (.26) | | 1.38 (0.38, 2.38) | 0.007 | 0.35 |
| Alabama Punishment | Baseline | 6.02 (.21) | 6.39 (.19) | |  |  |  |
|  | 6-week | 5.44 (.17) | 5.62 (.16) | | -0.21 (-0.78, 0.36) | 0.47 | -0.08 |
|  | 3 months | 5.46 (.14) | 5.50 (.13) | | -0.33 (-0.89, 0.22) | 0.23 | -0.13 |
| PSC Attention Problems | Baseline | 3.97 (.18) | 4.51 (.17) | |  |  |  |
|  | 6-week | 3.44 (.17) | 3.83 (.17) | | -0.20 (-0.78, 0.38) | 0.50 | -0.09 |
|  | 3 months | 3.15 (.16) | 3.49 (.15) | | -0.24 (-0.81, 0.33) | 0.41 | -0.02 |
| PSC Internalising | Baseline | 3.24 (.13) | 3.35 (.11) | |  |  |  |
|  | 6-week | 2.83 (.12) | 2.98 (.12) | | 0.04 (-0.37, 0.45) | 0.84 | 0.03 |
|  | 3 months | 2.85 (.11) | 3.01 (.11) | | 0.05 (-0.37, 0.46) | 0.82 | 0.04 |
| PSC Exernalising | Baseline | 3.63 (.12) | 3.66 (.13) | |  |  |  |
|  | 6-week | 3.30(.11) | 7.32 (.11) | | -0.01 (-0.46, 0.44) | 0.96 | -0.01 |
|  | 3 months | 3.20 (.11) | 3.37 (.10) | | 0.15 (-0.27, 0.56) | 0.48 | 0.11 |

Abbreviations. EUC = Enhanced usual care; LS = Least Square; HSCL = Hopkins Symptom Checklist (depression subscale score range: 10-40; anxiety subscale score range: 15-60;

higher scores indicate elevated anxiety or depression); WHODAS = WHO Disability Assessment Schedule (total score range: 0-48; higher scores indicate more severe impairment);

PCL-5 = Posttraumatic Stress Disorder Checklist (total score range: 0-80; higher scores indicate more severe PTSD severity); PSYCHLOPS = Psychological Outcomes Profiles (total

score range: 0-20; higher scores indicate poorer outcome); PG-13 = Prolonged Grief Disorder 13 (total score range: 11-57; higher scores indicate poorer outcome). Alabama Parenting

Questionnaire (Parental Involvement subscale score range: 10-50; Positive Parent subscale score range: 6-30; Supervision subscale score range 10-50; Discipline subscale score range

6-30; Punishment subscale score range 3-15; higher scores indicate elevated parental involvement, positive parenting, supervision, discipline, and punishment). Pediatric Symptom

Checklist is child’s self-report (PSC; Attention Problems subscale score range: 0-10; Internalising subscale score range: 0-10; Externalising subscale score range: 0-14). Effect size was

calculated by the difference in least square means between intervention and EUC from mixed model divided by the pooled standard deviation.
