# Supplementary material for: Effectiveness of a brief group behavioral intervention for common mental disorders in Syrian refugees in Jordan: A randomized controlled trial
Source: PLoS Med. 2022 Mar 17;19(3):e1003949. doi: 10.1371/journal.pmed.1003949 (PMC8929659; doi:10.1371/journal.pmed.1003949)
Supplement: S4 Table — (DOCX) [file pmed.1003949.s006.docx]

S4 Table. Summary statistics and results from mixed model analysis of primary and secondary outcomes controlling for

trauma exposure and post-migration living difficulties

|  | | Descriptive statistics | | Mixed model analysis | | | |
| --- | --- | --- | --- | --- | --- | --- | --- |
| Primary and secondary outcomes | Visit | gPM+ (n = 204) | EUC (n = 206) | | Difference in LS mean (95%CI) | P-value | Effect size^a^ |
|  |  | Estimated Mean (SE) | Estimated Mean (SE) | |  |  |  |
| HSCL-25 Depression | Baseline (n = 410) | 36.35 (.60) | 35.33 (.60) | |  |  |  |
|  | 6-week (n = 367) | 29.78 (.72) | 32.62 (.69) | | 4.24 (2.26, 5.86) | .001 | 0.92 |
|  | 3 months (n -= 357) | 28.82 (.70) | 31.49 (.67) | | 4.06 (1.90, 5.48) | .001 | 0.90 |
| HSCL-25 Anxiety | Baseline (n = 410) | 24.71 (.42) | 25.08 (.42) | |  |  |  |
|  | 6-week (n = 367) | 20.31 (.50) | 22.00 (.48) | | 1.53 (0.11, 2.96) | .03 | 0.20 |
|  | 3 months (n = 357) | 19.93 (.50) | 19.74 (.47) | | -0.36 (-0.08, 2.77) | .64 | -0.12 |
| WHODAS | Baseline (n = 410) | 23.60 (.35) | 23.86 (.35) | |  |  |  |
|  | 6-week (n = 366) | 14.76 (.56) | 15.51 (.58) | | 0.38 (-1.28, 2.04) | .66 | 0.26 |
|  | 3 months (n = 357) | 15.80 (.53) | 14.85 (.53) | | -1.32 (-2.91, 0.27) | .10 | -0.484 |
| PCL-5 | Baseline (n = 410) | 25.50 (.87) | 27.71 (.87) | |  |  |  |
|  | 6-week (n = 366) | 15.71 (1.06) | 17.95 (1.01) | | 1.43 (-1.87, 4.75) | 0.39 | 0.20 |
|  | 3 months (n = 357) | 9.83 (0.97) | 10.75 (.92) | | 0.12 (-3.20, 3.44) | 0.95 | 0.02 |
| PSYCHLOPS | Baseline (n = 410) | 16.46 (.27) | 15.74 (.27) | |  |  |  |
|  | 6-week (n = 365) | 13.32 (.36) | 13.69 (.35) | | 1.09 (0.19, 1.98) | 0.02 | 0.57 |
|  | 3 months (n = 357) | 13.44 (.34) | 13.61 (.33) | | 1.15 (0.25, 2.04) | .01 | 0.60 |
| ‏PG-13 | Baseline (n = 234) | 27.91 (.91) | 29.26 (.94) | |  |  |  |
|  | 6-week (n = 207) | 26.79 (1.05) | 27.88 (1.06) | | -0.25 (-3.50, 3.00) | 0.88 | -0.05 |
|  | 3 months (n = 202) | 20.41 (.76) | 21.52 (.76) | | -0.25 (-3.12, 2.60) | 0.84 | -0.05 |
| PQ | Baseline (n = 410) | 13.31 (.19) | 13.38 (.19) | |  |  |  |
|  | 6-week (n = 366) | 14.86 (.16) | 14.41 (.15) | | -0.60 (-1.25, 0.05) | 0.07 | -0.43 |
|  | 3 months (n = 357) | 15.11 (.14) | 14.92 (.13) | | -0.33 (-0.96, 0.29) | 0.30 | -0.24 |
| Alabama Involvement | Baseline (n = 400) | 34.82 (.61) | 34.67 (.61) | |  |  |  |
|  | 6-week (n = 359) | 33.75 (.66) | 33.01 (.64) | | -0.46 (-2.55, 1.63) | 0.66 | -0.11 |
|  | 3 months (n = 352) | 31.93 (.65) | 31.89 (.61) | | 0.24 (-1.96, 2.44) | 0.80 | 0.06 |
| Alabama Supervision | Baseline (n = 408) | 14.93 (.33) | 14.80 (.33) | |  |  |  |
|  | 6-week (n = 364) | 12.87 (.31) | 13.57 (.30) | | 0.85 (-0.28, 1.98) | 0.14 | 0.35 |
|  | 3 months (n = 354) | 12.46 (.24) | 12.46 (.24) | | 0.22 (-0.83, 1.27) | 0.68 | 0.09 |
| Alabama Positive Parenting | Baseline (n = 407) | 24.05 (.34) | 24.66 (.34) | |  |  |  |
|  | 6-week (n = 362) | 23.45 (.36) | 23.63 (.35) | | -.42 (-1.58, 0.73) | 0.46 | -.18 |
|  | 3 months (n = 352) | 21.86 (.35) | 22.32 (.33) | | -.14 (-1.29, 1.01) | 0.79 | -.06 |
| Alabama Discipline | Baseline (n = 406) | 15.43 (.27) | 14.81 (.27) | |  |  |  |
|  | 6-week (n = 364) | 13.55 (.28) | 13.59 (.26) | | 0.74 (-0.29, 1.76) | 0.16 | 0.37 |
|  | 3 months (n = 352) | 132.94 (.27) | 13.64 (.26) | | 1.40 (0.43, 2.37) | 0.005 | 0.70 |
| Alabama Punishment | Baseline (n = 410) | 6.04 (.18) | 6.37 (.18) | |  |  |  |
|  | 6-week (n = 365) | 5.46 (.16) | 5.58 (.16) | | -0.14 (-0.69, 0.41) | 0.61 | -0.11 |
|  | 3 months (n = 356) | 5.44 (.14) | 5.51 (.13) | | -0.20 (-0.73, 0.34) | 0.47 | -0.32 |
| PSC Attention Problems | Baseline (n = 374) | 3.87 (.16) | 9.47 (.16) | |  |  |  |
|  | 6-week (n = 322) | 3.41 (.17) | 8.80 (.17) | | -0.19 (-0.76, 0.38) | 0.52 | -0.17 |
|  | 3 months (n = 312) | 3.13 (.16) | 8.49 (.15) | | -0.21 (-0.78, 0.36) | 0.46 | -0.19 |
| PSC Internalising | Baseline (n = 373) | 3.22 (.11) | 8.36 (.11) | |  |  |  |
|  | 6-week (n = 318) | 2.83 (.12) | 7.98 (.12) | | 0.04 (-0.36, 0.44) | 0.85 | 0.06 |
|  | 3 months (n = 305) | 2.85 (.11) | 8.01 (.11) | | 0.06 (-0.35, 0.45) | 0.79 | 0.09 |
| PSC Externalising | Baseline (n = 372) | 3.62 (.11) | 3.65 (.12) | |  |  |  |
|  | 6-week (n = 322) | 3.29(.11) | 3.32 (.11) | | 0.01 (-0.43, 0.42) | 0.99 | 0.01 |
|  | 3 months (n = 311) | 3.18 (.11) | 3.38 (.11) | | 0.18 (-0.22, 0.57) | 0.38 | 0.2 |
|  |  |  |  | |  |  |  |

Abbreviations. EUC = Enhanced usual care; LS = Least Square; HSCL = Hopkins Symptom Checklist (depression subscale score range: 10-40; anxiety subscale score range: 15-60;

higher scores indicate elevated anxiety or depression); WHODAS = WHO Disability Assessment Schedule (total score range: 0-48; higher scores indicate more severe impairment);

PCL-5 = Posttraumatic Stress Disorder Checklist (total score range: 0-80; higher scores indicate more severe PTSD severity); PSYCHLOPS = Psychological Outcomes Profiles (total

score range: 0-20; higher scores indicate poorer outcome); PG-13 = Prolonged Grief Disorder 13 (total score range: 11-57; higher scores indicate poorer outcome). PQ = Prodromal

Questionnaire (total score range: 0-64; higher scores indicate poorer outcome Alabama Parenting Questionnaire (Parental Involvement subscale score range: 10-50; Positive Parent

subscale score range: 6-30; Supervision subscale score range 10-50; Discipline subscale score range 6-30; Punishment subscale score range 3-15; higher scores indicate elevated

parental involvement, positive parenting, supervision, discipline, and punishment). Pediatric Symptom Checklist is child’s self-report (PSC; Attention Problems subscale score range:

0-10; Internalising subscale score range: 0-10; Externalising subscale score range: 0-14). Effect size was calculated by the difference in least square means between intervention

and EUC from mixed model divided by the pooled standard deviation.
