# Supplementary material for: Effectiveness of a brief group behavioral intervention for common mental disorders in Syrian refugees in Jordan: A randomized controlled trial
Source: PLoS Med. 2022 Mar 17;19(3):e1003949. doi: 10.1371/journal.pmed.1003949 (PMC8929659; doi:10.1371/journal.pmed.1003949)
Supplement: S5 Table — (DOCX) [file pmed.1003949.s007.docx]

S5 Table. Summary statistics and results from mixed model analysis of primary and secondary outcomes for

participants with probable mental disorder (defined as mean item score of ≥2 on the Hopkins Symptom Checklist)

|  | | Descriptive statistics | | Mixed model analysis | | |
| --- | --- | --- | --- | --- | --- | --- |
| Primary and secondary outcomes | Visit | gPM+ (n = 184) | EUC (n = 181) | Difference in LS mean (95%CI) | P-value | Effect size^a^ |
|  |  | Estimated Mean (SE) | Estimated Mean (SE) |  |  |  |
|  | Baseline | 38.24 (.62) | 37.24 (.62) | HSCL-25 Depression |  |  |
|  | 6-week | 31.46 (.80) | 33.72 (.76) | 3.74 (1.61, 5.88) | .001 | 0.42 |
|  | 3 months | 30.11 (.78) | 33.06 (.74) | 4.43 (2.44, 6.42) | .001 | 0.49 |
| HSCL-25 Anxiety | Baseline | 26.38 (.40) | 26.76 (.40) |  |  |  |
|  | 6-week | 21.38 (.55) | 22.73 (.52) | 0.98 (-0..59, 2.54) | .62 | 0.16 |
|  | 3 months | 20.60 (.55) | 20.62 (.52) | -.36 (-2.04, 1.32) | .68 | -0.06 |
| WHODAS | Baseline | 23.71 (.39) | 24.09 (.39) |  |  |  |
|  | 6-week | 14.45 (.60) | 15.37 (.62) | 0.54 (-1.24, 2.33) | .55 | 0.11 |
|  | 3 months | 16.02 (.58) | 15.14 (.59) | -1.27 (-3.02, 0.49) | .16 | -0.25 |
| PCL-5 | Baseline | 27.70 (1.09) | 28.93 (1.08) |  |  |  |
|  | 6-week | 17.49 (1.18) | 19.06 (1.12) | 0.33 (-3.40, 4.05) | 0.86 | 0.02 |
|  | 3 months | 10.92 (1.17) | 11.39 (1.11) | -.76 (-4.46, 2.94) | 0.69 | -0.05 |
| PSYCHLOPS | Baseline | 16.69 (.29) | 15.95 (.27) |  |  |  |
|  | 6-week | 13.36 (.37) | 13.94 (.36) | 0.84 (-.14, 1.83) | 0.09 | 0.22 |
|  | 3 months | 13.60 (.36) | 13.87 (.35) | 0.94 (0.04, 1.83) | .04 | 0.25 |
| ‏PG-13 | Baseline | 28.81 (1.00) | 29.06 (1.03) |  |  |  |
|  | 6-week | 27.57 (1.16) | 28.40 (1.17) | .58 (-2.94, 4.11) | 0.74 | 0.06 |
|  | 3 months | 20.97 (.84) | 21.64 (.83) | 0.42 (-2.67, 3.52) | 0.79 | 0.04 |
| PQ | Baseline | 12.97 (.23) | 13.24 (.23) |  |  |  |
|  | 6-week | 14.73 (.18) | 14.25 (.17) | -0.75 (-1.49, 0.01) | 0.05 | -0.27 |
|  | 3 months | 14.99 (.16) | 14.84 (.16) | -0.41 (-01.13, 0.30) | 0.26 | -0.15 |
| Alabama Involvement | Baseline | 34.59 (.67) | 34.25 (.66) |  |  |  |
|  | 6-week | 33.70 (.73) | 32.49 (.70) | -0.87 (-3.15, 1.42) | 0.46 | -0.10 |
|  | 3 months | 31.74 (.70) | 31.85 (.66) | 0.45 (-1.95, 2.88) | 0.71 | 0.05 |
| Alabama Supervision | Baseline | 15.33 (.38) | 14.67 (.38) |  |  |  |
|  | 6-week | 13.06 (.34) | 13.51 (.32) | 1.11 (-.15, 2.36) | 0.08 | 0.23 |
|  | 3 months | 12.54 (.29) | 12.51 (.27) | 0.63 (-0.54, 1.82) | 0.29 | 0.13 |
| Alabama Positive Parenting | Baseline | 23.96 (.37) | 24.80 (.37) |  |  |  |
|  | 6-week | 23.46 (.41) | 23.42 (.39) | -0.86 (-2.12, 0.39) | 0.18 | -0.18 |
|  | 3 months | 21.49 (.37) | 22.34 (.35) | 0.02 (-1.20, 1.24) | 0.97 | 0.00 |
| Alabama Discipline | Baseline | 15.87 (.32) | 14.99 (.32) |  |  |  |
|  | 6-week | 13.82 (.30) | 13.64 (.29) | 0.75 (-0.38, 1.89) | 0.91 | 0.19 |
|  | 3 months | 13.11 (.30) | 13.72 (.29) | 1.53 (0.46, 2.61) | 0.005 | 0.38 |
| Alabama Punishment | Baseline | 6.31 (.21) | 6.39 (.21) |  |  |  |
|  | 6-week | 5.60 (.18) | 5.65 (.17) | -0.02 (-0.64, 0.60) | 0.94 | -0.01 |
|  | 3 months | 5.60 (.16) | 5.56 (.15) | -0.12 (-0.71, 0.47) | 0.69 | -0.05 |
| PSC Attention Problems | Baseline | 3.94 (.17) | 4.47 (.16) |  |  |  |
|  | 6-week | 3.62 (.18) | 3.87 (.18) | -0.39 (-1.02, 0.25) | 0.23 | -0.18 |
|  | 3 months | 3.30 (.18) | 3.59 (.16) | -0.33 (-0.96, 0.30) | 0.30 | -0.15 |
| PSC Internalizing | Baseline | 3.24 (.12) | 3.40 (.11) |  |  |  |
|  | 6-week | 2.90 (.13) | 3.04 (.13) | -0.02 (-0.46, 0.42) | 0.92 | 0.01 |
|  | 3 months | 3.01 (.13) | 3.11 (.12) | -0.05 (-0.51, 0.41) | 0.82 | -0.21 |
| PSC Exernalizing | Baseline | 3.72 (.13) | 3.63 (.12) |  |  |  |
|  | 6-week | 3.39 (.12) | 3.30 (.12) | -0.01 (-0.48, 0.47) | 0.98 | 0.01 |
|  | 3 months | 3.26 (.12) | 3.44 (.12) | 0.27 (-0.17, 0.71) | 0.23 | 0.17 |

Abbreviations. EUC = Enhanced usual care; LS = Least Square; HSCL = Hopkins Symptom Checklist (depression subscale score range: 10-40;anxiety subscale score range: 15-60; higher

scores indicate elevated anxiety or depression); WHODAS = WHO Disability Assessment Schedule (total score range: 0-48; higher scores indicate more severe impairment); PCL-5 =

Posttraumatic Stress Disorder Checklist (total score range: 0-80; higher scores indicate more severe PTSD severity); PSYCHLOPS = Psychological Outcomes Profiles (total score range: 0-20;

higher scores indicate poorer outcome); PG-13 = Prolonged Grief Disorder 13 (total score range: 11-57; higher scores indicate poorer outcome). Alabama Parenting Questionnaire (Parental Involvement subscale score range: 10-50; Positive Parent subscale score range: 6-30; Supervision subscale score range 10-50; Discipline subscale score range 6-30; Punishment subscale

score range 3-15; higher scores indicate elevated parental involvement, positive parenting, supervision, discipline, and punishment). Pediatric Symptom Checklist (PSC; Attention Problems

subscale score range: 0-10; Internalizing subscale score range: 0-10; Externalizing subscale score range: 0-14). Effect size was calculated by the difference in least square means between

intervention and EUC from mixed model divided by the pooled standard deviation.
