# Supplementary material for: Effectiveness of a brief group behavioral intervention for common mental disorders in Syrian refugees in Jordan: A randomized controlled trial
Source: PLoS Med. 2022 Mar 17;19(3):e1003949. doi: 10.1371/journal.pmed.1003949 (PMC8929659; doi:10.1371/journal.pmed.1003949)

S1 Fig. Path Model of Relationship Between Group Problem Management Plus (gPM+)/Enhanced Usual Care (EUC), Change in Parenting Style, and Change in Children’s Externalizing Problems. Path model demonstrates that there was a significant relationship between gPM+ and reductions in disciplinary parenting (as measured by the Alabama Parenting Questionnaire) but no association between parenting style and externalising problems (as measured by the Pediatric Symptom Checklist). Value are unstandardized coefficients (standard error). * significant paths.


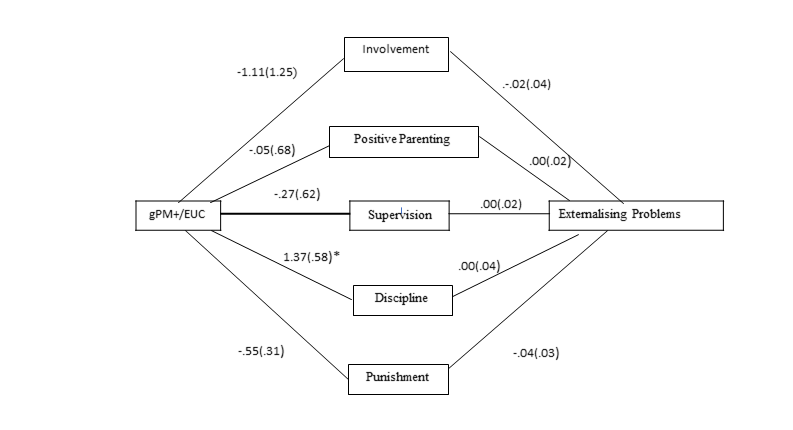

Supplement: S1 Fig — Path model demonstrates that there was a significant relationship between gPM+ and reductions in disciplinary parenting (as measured by the APQ) but no association between parenting style and externalizing problems (as measured by the PSC). Value are unstandardized coefficients (standard error). *Significant paths. APQ, Alabama Parenting Questionnaire; EUC, enhanced usual care; gPM+, Group Problem Management Plus; PSC, Pediatric Symptoms Checklist. (DOCX) [file pmed.1003949.s008.docx]
